# Challenges and the Way forward in Diagnosis and Treatment of Tuberculosis Infection

**DOI:** 10.3390/tropicalmed8020089

**Published:** 2023-01-28

**Authors:** Kai Ling Chin, Luis Anibarro, Maria E. Sarmiento, Armando Acosta

**Affiliations:** 1 Faculty of Medicine and Health Sciences, Universiti Malaysia Sabah, Kota Kinabalu 88400, Malaysia; 2 Borneo Medical and Health Research Centre, Faculty of Medicine and Health Sciences, Universiti Malaysia Sabah, Kota Kinabalu 88400, Malaysia; 3 Tuberculosis Unit, Infectious Diseases and Internal Medicine Department, Complexo Hospitalario Universitario de Pontevedra, 36071 Pontevedra, Spain; 4 Immunology Research Group, Galicia Sur Health Research Institute (IIS-GS), 36312 Vigo, Spain; 5 School of Health Sciences, Universiti Sains Malaysia, Health Campus, Kubang Kerian 16150, Malaysia

**Keywords:** latent tuberculosis, multidrug-resistant tuberculosis, diagnosis, treatment, host markers, *Mycobacterium tuberculosis*-specific antigens

## Abstract

Globally, it is estimated that one-quarter of the world’s population is latently infected with *Mycobacterium tuberculosis* (Mtb), also known as latent tuberculosis infection (LTBI). Recently, this condition has been referred to as tuberculosis infection (TBI), considering the dynamic spectrum of the infection, as 5–10% of the latently infected population will develop active TB (ATB). The chances of TBI development increase due to close contact with index TB patients. The emergence of multidrug-resistant TB (MDR-TB) and the risk of development of latent MDR-TB has further complicated the situation. Detection of TBI is challenging as the infected individual does not present symptoms. Currently, there is no gold standard for TBI diagnosis, and the only screening tests are tuberculin skin test (TST) and interferon gamma release assays (IGRAs). However, these tests have several limitations, including the inability to differentiate between ATB and TBI, false-positive results in BCG-vaccinated individuals (only for TST), false-negative results in children, elderly, and immunocompromised patients, and the inability to predict the progression to ATB, among others. Thus, new host markers and Mtb-specific antigens are being tested to develop new diagnostic methods. Besides screening, TBI therapy is a key intervention for TB control. However, the long-course treatment and associated side effects result in non-adherence to the treatment. Additionally, the latent MDR strains are not susceptible to the current TBI treatments, which add an additional challenge. This review discusses the current situation of TBI, as well as the challenges and efforts involved in its control.

## 1. Introduction

Tuberculosis (TB) has remained an undefeatable disease for more than a century. In 2020, 10 million new TB cases and over 1.3 million deaths were reported [1]. Drug-resistant TB is one of the main challenges of the TB pandemic [2,3]. It is estimated that half a million new multidrug-resistant TB (MDR-TB) and extensively drug-resistant TB (XDR-TB) cases appear every year [2,3]. Only 57% of patients with MDR-TB who receive the treatment recommended by WHO were cured [2,4]. MDR-TB is increasing at an annual rate of >20 percent; hence, it will contribute to the estimated 31.8 million TB deaths that will be produced over the period of 2020–2050 [5]. Other estimates calculated that over the next 35 years, MDR-TB will kill 75 million people and could cost the global economy a cumulative $16.7 trillion [6].

TBI, formerly known as LTBI, is a state where a persistent immune response to stimulation Mtb antigens (Ags) is demonstrated without the presence of clinical signs and symptoms of manifested active tuberculosis (ATB) [7]. Among the TBI individuals, 5 to 10% will develop ATB [4]. Globally, it is estimated that one-quarter of the world’s population has TBI [8], representing an important reservoir for future TB cases, mainly in risk groups such as the elderly, children, patients with human immunodeficiency virus (HIV), diabetes, primary immunodeficiency, cancer, and under immunosuppressive treatment, among others, some of them infected with MDR-TB [9,10]. It was reported that the South-East Asia, Western-Pacific, and Africa regions had the highest prevalence of TBI, accounting for around 80% in 2014, where China had the highest estimated LTBI burden with 350 million infections, followed by India with around 120 million infections [11]. It is estimated that three in every 1000 people globally carry a latent MDR-TB infection and are at risk to develop MDR-TB, so prevention of new MDR-TB cases from this growing reservoir is a priority [12].

A study by Reichler et al. (2020) showed that the main predisposing factors or the likelihood for acquiring TBI are close contact proximity with an index patient with cavitation in both left and right lungs and positive-sputum smear, sharing a bedroom with an index patient within a household, exposure of more than 250 h, and exposure to more than one index patient [13]. Among the high-risk factors that significantly increase the risk of progression from TBI to ATB are HIV infection, organ transplantation, silicosis, use of tumor necrosis factor-alpha blockers, close contact with TB patients within the past 2 years, and chronic renal failure (end-stage renal disease, ESRD) under dialysis treatment [10,14]. Medium-risk factors include fibronodular lung diseases, immigration from high TB burden countries, healthcare workers, prisoners, and homeless persons; while low-risk factors include diabetes mellitus, smoking, corticosteroids use, and underweight/malnutrition [10,14]. 

Although TB incidence showed a decrease of about 2% per year between 2015 and 2019, with a cumulative reduction of 9%, this trend, if maintained, will not allow us to meet the milestone of the End TB Strategy by 2050, which is set at a 20% reduction [4]. It is hardly possible to create a world free of TB unless progression to ATB is prevented, underlining the need for TBI screening and preventive therapy as key interventions for TB control. 

Here, we will review the common modalities for TBI screening and their applications in testing the high-risk populations, including the children, elderly, immunocompromised individuals, healthcare workers, and immigrants and travelers from high-endemic TB countries, among others. Additionally, new host- and Mtb-derived biomarkers to be used for detection of TBI are discussed. Additionally, issues related to non-adherence and resistance in TBI treatment and new trial programs for MDR-TB strains in TBI are reviewed.

## 2. Screening Tests for TBI

**Figure 1:**
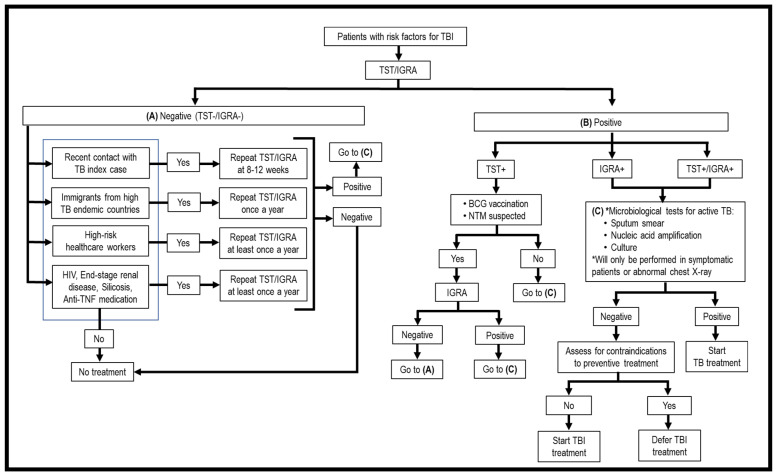
Flowchart of screening for TBI.

As TBI is asymptomatic, the first issue to be considered is who should be screened for TBI. Generally, high-risk groups fall into two categories, i.e., persons with recent exposure to Mtb and persons with immunosuppressive treatment or medical conditions that weaken the immune system. These patients should be screened for Mtb infection. Once a risk individual is identified, the second issue is what screening modalities should be used to detect TBI. Currently, only two methods have been approved for TBI screening, i.e., tuberculin skin test (TST) and interferon-gamma release assays (IGRAs) (Figure 1, Table 1) [15]. They are “indirect tests” as they do not detect the bacilli directly, but instead evaluate the cell-mediated immune response/reactivity of the T lymphocytes toward Mtb Ags. These tests cannot discriminate TBI from ATB, recently acquired and old infections, and cannot monitor the clinical efficacy of TBI treatment; thus, it is necessary to perform clinical assessments and laboratory diagnostic studies for ATB once TST/IGRA is positive (Figure 1, Table 1). Another issue is how frequently a suspected TBI individual should be screened. A one-time screening is recommended for persons who are at low risk for TB exposure, and annual screening among persons who are at continued risk of exposure [16]. Evaluating the accuracy of TST and IGRAs in diagnosing TBI remains a problem since there is no “gold standard”. Persistently, TST/IGRA negative individuals were detected among highly Mtb-exposed healthcare workers, household contacts, and gold miners living and working in close quarters in contact with ATB cases. These individuals are dubbed as “resisters”, meaning resistance to primary infection and TBI [17,18]. Due to the lack of accuracy of current diagnostic methods for TBI, here we will also review new modalities that have been tested/developed for screening of TBI.

### 2.1. Skin Tests

Tuberculin skin test (TST) is also known as the Mantoux test. It is performed by injecting 0.1 mL (5 IU) of tuberculin purified protein derivative (PPD) intradermally into the forearm. After 48 to 72 h, the delayed-type hypersensitivity (DTH) response is monitored by measuring the diameter of the induration (firm swelling due to inflammation) at the site of injection by a trained health professional [23]. The induration measurement result should be interpreted based on the patient’s history and risk of developing ATB, with cut-offs set at ≥5 mm of induration in HIV patients, immunosuppressed individuals, or recent close contacts; ≥10 mm of induration in immigrants from high TB burden countries, drug users, residents and employees of high-risk congregate settings (e.g., prisons, nursing homes, hospitals, homeless shelters, etc.), or mycobacteriology laboratory workers; and ≥15 mm for a person with no risk factors for TB [23,24] (Table 2). 

A positive TST reaction could be due to ATB, TBI, or a previously resolved infection with Mtb [23]. Therefore, a medical evaluation based on TB history, physical examination, radiography, and, if necessary, microbiological tests are indicated to rule out ATB before prescribing TBI treatment. A current concern of the test is that a false-positive response can be produced due to vaccination with the live attenuated *M. bovis* bacillus Calmette-Guérin (BCG) vaccine, or infection with non-tuberculous mycobacteria (NTM) as the tuberculin used in TST is a mixture of a large number of Mtb Ags, which are also present in other mycobacteria [23]. Hence, to avoid these non-specific responses, individual more specific proteins from *Mycobacterium tuberculosis* complex (MTBC) members and Mtb have been tested for their ability to induce DTH responses [25,26]. These Ags could serve as the next-generation PPD candidates. Nonetheless, it should be noted that skin tests, more specific than TST, are not yet available for everyday clinical practice. Additionally, all skin tests use the same intradermal injection procedure, subjective interpretation, and lack of positive or negative controls, making it possible to have false-negative results, for example in immunocompromised people.

Early secretory antigenic 6 kDa (ESAT-6) and culture filtrate protein 10 kDa (CFP-10) are Mtb highly specific region of difference 1 (RD1)-encoded proteins that are not present in BCG and most of NTM. A study in guinea pigs showed that skin tests with these antigens induced strong DTH responses only in Mtb-infected guinea pigs, but not in BCG-vaccinated, *M. avium*-sensitized, and control groups [25]. Although PPD had high sensitivity in the diagnosis of TB (82%) compared to a combination of ESAT-6 and CFP-10 Ags (73%), the latter had a higher specificity of 93% compared to 7% in PPD [25]. A study on guinea pigs also showed the possibility to use this combination of Ags as a prognostic skin test to predict the risk of developing ATB based on the observation that larger skin test responses correlated with a shorter time of active infection presentation, and vice versa [27].

A first human trial with intradermal recombinant dimer ESAT-6 (rdESAT-6) in 2008 showed that the response to 0.1 µg of rdESAT-6 was similar to tuberculin, the induration only caused transient redness after 24 h with no serious side effects and did not cause sensitization, suggesting its potential as a skin test reagent [28]. In 2010, further investigation with rdESAT-6 and recombinant CFP-10 (rCFP-10) in combination showed that this skin test reagent was safe and non-sensitizing as well [29]. In 2012, Diaskintest^®^ was developed (containing recombinant CFP-10 and ESAT-6) [30]. This test had high sensitivity for PTB (97.3% in children and adolescents, 84.2% in adults), EPTB (89.7%) and TBI (94.9%), and high specificity in BCG-vaccinated (100%), pulmonary non-TB diseases (94.6%), and extrapulmonary non-TB diseases (98.5%) [30]. A specificity study in NTM-infected patients showed negative responses in *M. avium*-, *M. xenopi*-, *M. fortuitum*-, and *M. chelonae*-infected patients, while positive responses were observed in *M. kansasii*-infected patients [31]. The efficiency of Diaskintest^®^ for TB screening in children and adolescents showed that it had comparable sensitivity with the Mantoux test and was suitable for mass screening as it showed high specificity under the conditions of mass BCG vaccination [32]. A study by Hoff et al. (2015) found that the sensitivity of these antigens would be reduced in HIV-infected patients with severe immunosuppression [33].

Other RD1 Ags, i.e., PE35 and PPE68, also induced strong DTH responses in Mtb-immunized guinea pigs, but not in BCG-vaccinated or in *M. avium*- and *M. vaccae*-infected groups. However, the responses were lower than with ESAT-6 and CFP-10 [34]. 

A study suggested that MPT64 induced stronger DTH responses compared to ESAT-6 and CFP-10 only in Mtb-infected guinea pigs, but not in BCG-vaccinated and control groups, and the combination of ESAT-6 and MPT64 Ags induced higher DTH responses than PPD [35]. However, MPT64 is considered a MTBC-specific Ag [36]. MPT64, MPT70, MPT63, and MTC28 induced 8 to 15 times stronger DTH responses in BCG-immunized guinea pigs than in *M. avium*-infected animals, while 19 kDa, MPT51, Ag85B, 38 kDa, MPT32, and KatG were non-specific Ags as DTH responses were observed in both BCG- and *M. avium*-immunized guinea pigs [36]. 

Rv2645, an RD13 protein, stimulated DTH responses that can differentiate between Mtb-infected mice from the BCG-immunized group [37]. Another protein that has been used as a stimulating Ag that showed specificity to TB infection was the Rv2654, which belongs to the RD11 region [38].

WHO has recently released a rapid communication: TB antigen-based skin tests for the diagnosis of TB infection using new Mtb antigen-based skin tests (TBST), i.e., C-Tb (Serum Institute of India, India), C-TST (formerly known as ESAT6-CFP10 test, Anhui Zhifei Longcom, China), and Diaskintest^®^ (Generium, Russian Federation). They conclude that TBSTs were found to be accurate for the detection of TB infection compared with IGRAs and TSTs, have a good safety profile, and may be cost-effective for the detection of Mtb infection [19].

### 2.2. Interferon-Gamma Release Assays (IGRAs)

IGRAs are based on the stimulation of blood lymphocytes with Mtb specific antigens, which induce the production of IFN-γ in individuals with previous contact with Mtb. A combination of ESAT-6 and CFP-10 Ags induces IFN-γ production by T cells sensitized to Mtb from individuals with previous contact with the bacteria, which supports their use in all the IGRAs alone or in combination with other Ags [39]. IGRAs are immunological assays that are not influenced by BCG vaccination or exposure to most NTM infections and are more robust than TST in their performance in immunocompromised patients as specific Mtb Ags are used for stimulating host T lymphocytes to produce IFN-γ [40]. The results of the test could be obtained within 24 h [41]. The accuracy of IGRAs for children aged <5 years old is still a matter of debate [41,42,43,44]. However, both TST and IGRA had similar limitations in differentiating ATB and TBI, differentiating recently acquired and old infections, or predicting the progression of TBI to ATB [45]. IGRAs should not be used to confirm or rule out ATB, particularly in high TB burden regions because false-negative IGRA results were reported in one-tenth of culture-confirmed pulmonary TB (PTB) cases [46].

In 2011, WHO recommended blood-based IGRAs including QuantiFERON^®^-TB Gold-In-Tube (QFT^®^-GIT; Qiagen, Hilden, Germany) and T-SPOT^®^.TB (Oxford Immunotec Ltd., Milton, UK) for the diagnosis of TBI. In recent years, many new IGRAs have been available commercially, including QuantiFERON^®^-TB Gold Plus (QFT^®^-Plus; Qiagen, Hilden, Germany), QIAreach^TM^ QuantiFERON^®^-TB (QIAreach^TM^; Qiagen, Hilden, Germany), Beijing Wantai’s TB-IGRA (Wantai; WanTai Biological Pharmacy Enterprise Co., Ltd., Beijing, China), Standard E TB-Feron ELISA (TBF; SD Biosensor, Suwon-si, Gyeonggi-do, Republic of Korea) and T-Cell Select^TM^ (Oxford Immunotec Ltd., Milton, UK). In 2022, WHO recommended that QFT^®^-Plus and Wantai to be used as alternative IGRAs as the available data showed that the performance of both tests is comparable to WHO-recommended IGRAs [20].

#### 2.2.1. QuantiFERON^®^-TB Gold-In Tube (QFT^®^-GIT), QuantiFERON^®^-TB Gold Plus (QFT^®^-Plus) and QIAreach^TM^ QuantiFERON^®^-TB (QIAreach^TM^)

QFT^®^-GIT and QFT^®^-Plus are methods based on evaluation by an enzyme-linked immunosorbent assay (ELISA) of the amount of INF-γ that is released upon stimulation of blood lymphocytes with highly specific Mtb Ags. The tests were approved by the U.S. Food and Drug Administration (FDA) in 2007 and 2017, respectively. QFT^®^-GIT was solely based on CD4 T cell responses to a combination of synthetic peptides from highly specific Mtb Ags (ESAT-6, CFP-10, and TB7.7) in a single tube. While the new generation QFT^®^-Plus has two tubes (TB1 and TB2) with different Ag cocktails, i.e., each tube consists of whole Mtb Ags (ESAT-6 and CFP-10), and tube TB2, in addition, has six short peptides that can also stimulate CD8 T cells. Theoretically, the presence and strength of the CD8 response may help the clinical assessment of patients with new or active infections as it is usually shut off in old and not active diseases [47]. Nonetheless, some studies have failed to confirm the relationship between a high CD8 response and recent Mtb infection [48].

Whole blood is used in QFT^®^-GIT and QFT^®^-Plus, which must be processed within 16 h of extraction. Each sample is inoculated into individual tubes (1 mL per tube) of Nil Control (NC), TB antigen (TBA), and Positive Control (PC; mitogen). The tubes are incubated at 37 °C for 16–20 h, followed by plasma collection for IFN-γ determination by ELISA [47] (Table 2). 

IFN-γ response using ESAT-6 and CFP-10 as single Ags was lower compared to Ag combinations, i.e., ESAT-6+CFP-10 and ESAT-6+CFP-10+TB7.7 [49]. However, IFN-γ response to ESAT-6+CFP-10+TB7.7 was not significantly greater than the response to ESAT-6+CFP-10 [49]. Thus, although QFT^®^-Plus uses two Ags instead of three in the QFT^®^-GIT version, sensitivity has not been globally affected [20,50]. Nonetheless, false-positive results were observed in low-risk patients, frequently with results close to the cut-off limits of the test [51]. Among healthcare workers with no identified risk factors and no history of TBI, a higher positivity rate was found with QFT^®^-Plus (3.0%) compared to QFT^®^-GIT (2.1%) [52]. 

A study by Nemes et al. (2017) showed that among individuals with QFT^®^-GIT IFN-γ values <0.2 IU/mL, 0.2–0.34 IU/mL, 0.35–0.7 IU/mL, and >0.7 IU/mL, TST positive results (≥5 mm) were 15%, 53%, 66%, and 91%, respectively [53]. Overall, 43% of individuals with IFN-γ values between 0.2 and 0.7 IU/mL had discordant results between QFT and TST, whereas 85% of concordance between <0.2 and >0.7 IU/mL was found [53]. Hence, deep attention has been given to the concept of the positivity threshold of QFT, defining the IFN-γ values ranging from 0.2 to 0.7 IU/mL as a “zone of uncertainty”. In follow-up samples, a high rate of conversions from negative to positive IGRA results occurred when the first QFT result was between 0.2–0.35 IU/mL, and a high rate of reversions of positive to negative IGRA results occur when the initial result was between 0.35–0.7 IU/mL, which had led to the proposal of the establishment of a borderline or equivocal range [54,55]. It is recommended that patients that fall in this zone during their first visit should repeat the QFT test to avoid over-treatment or under-treatment [55]. Another study recommended a borderline range of 0.2–0.99 IU/mL for a follow-up sample [56]. It is recommended that individuals that fall in this zone on their first visit should repeat the test after 2 weeks or it should be considered positive if the patient comes from a TB endemic country, is exposed to TB, or had chest X-ray with scars on the upper lobes. It should be noted that, in the same way it happens with TST, QFT poses a window period of about 8 weeks after exposure to Mtb [57,58].

QIAreach^TM^ QuantiFERON^®^-TB has been commercialized since 2021. It uses the same antigens in the TB2 tube of QFT^®^-Plus, but without the need to perform ELISA after incubation. The stimulated sample is placed on a lateral flow, digital detection cartridge named eStick, which is inserted into a battery-operated portable device named eHub for the detection of IFN-γ via nanoparticle fluorescence. Eight tests could be performed in a single run in 3–20 min, providing qualitative positive or negative results [59,60]. This assay shows comparable results with QFT^®^-Plus [59,60,61] and is suitable to be implemented in high-prevalence, low-resource settings [62]. Further studies are needed to accurately evaluate its performance in different populations, particularly in immunocompromised patients, children, and people living with HIV [61].

#### 2.2.2. T-SPOT^®^.TB and T-Cell Select^TM^

T-SPOT^®^.TB, approved by the U.S. Food and Drug Administration (FDA) in 2008, is an enzyme-linked immunospot-based assay (ELISPOT) that measures the number of cells that release IFN-γ upon stimulation with Mtb Ags [63]. Whole blood samples must be processed within 8 h, or within 32 h if T-Cell Xtend^®^ (Oxford Immunotec Ltd., Milton, UK) reagent is added. Peripheral blood mononuclear cells (PBMCs) need to be collected with FicollPaque^®^ (GE Healthcare, Chicago, IL, USA), and 250,000 ± 50,000 of PBMCs are seeded into each well of Nil Control (NC), Panel A (PA) (ESAT-6), Panel B (PB) (CFP-10), and Positive Control (PC) (mitogen). The plate is incubated at 37 °C with 5% carbon dioxide (CO_2_) for 16–20 h, and the IFN-γ released from the cells is determined by ELISPOT [63] (Table 2). 

A study by Janssens et al. (2007) showed that the number of spot-forming units (sfu) in culture-proven TB patients (107 ± 56) was higher than in TBI patients with a TST > 5 mm (54 ± 60) and TBI patients with a TST ≤ 5 mm (19 ± 27). At a threshold of 49.5 sfu, T-SPOT^®^.TB had a sensitivity of 83% and specificity of 74% in distinguishing TBI from ATB [64]. The consideration of the borderline T-SPOT^®^ results has demonstrated its utility to increase the test resolution [65].

T-Cell Select^TM^, manufactured since 2018 and approved by the FDA in 2022, is a reagent that enables the whole blood (stored at room temperature) to be processed within 54 h after collection. Additionally, it uses a magnetic bead-based system to automatically isolate PBMCs without negatively impacting the T cell function and the performance of T-SPOT^®^ [66].

#### 2.2.3. Beijing Wantai’s TB-IGRA (Wantai)

Wantai has been manufactured since 2011 and has been approved by the State Food and Drug Administration of China (CFDA). It contains three tubes, i.e., NC, PC, and TB-specific (containing a ESAT-6 and CFP-10 fusion protein) tubes. After 22 h of incubation, the IFN-γ level is quantified by ELISA [20,67]. According to the manufacturer product information sheet, Wantai has high specificity without interference from BCG vaccination and it showed superior performance compared to QFT^®^-Gold [67].

#### 2.2.4. TS-SPOT 

TS-SPOT (Tongsheng Biotech, Beijing, China) contains ESAT-6, CFP-10, and an additional RD-1 Ag, Rv3615c, which is broadly recognized by CD4^+^ and CD8^+^ T cells. TS-SPOT results had excellent concordance with T-SPOT^®^.TB (kappa value of 0.91) [68]. The addition of Rv3615c increased the sensitivity of TS-SPOT in the diagnosis of ATB (80.00% vs. 76.77%), but decreased its specificity (83.45 vs. 85.52%). This assay is cost-effective in low-income settings compared to current available IGRAs and has been proposed for its use in low-income/high-incidence settings [68]. 

#### 2.2.5. LIOFeron^®^TB/LTBI 

LIOFeron^®^TB/LTBI (Lionex GmbH, Braunschweig, Germany) consists of two tubes containing Mtb Ags, namely TB-A (ESAT-6, CFP-10, and TB7.7) and TB-B (alanine dehydrogenase (Ala-DH)). Ala-DH is a Mtb-specific Ag as it is not present in the BCG strains and is recognized by CD8^+^ T cells [69]. LIOFeron^®^TB/LTBI demonstrated concordance in sensitivity and specificity with QFT^®^-Plus in the diagnosis of ATB (90%, 98% vs. 98%, 97%) and TBI (94%, 97% vs. 85%, 94%) [69]. The high sensitivity of LIOFeron^®^TB/LTBI (94%) for the TBI group was based on the results of tube TB-B, suggesting that Ala-DH had higher sensitivity than ESAT-6 and CFP-10 for TBI [69]. 

### 2.3. Comparison of TST, QFT^®^, and T-SPOT^®^.TB in Screening of TBI among Different Populations

The American Thoracic Society, Infectious Diseases Society of America, and Centers for Disease Control and Prevention (ATS/IDSA/CDC) have recommended screening tests using either TST or IGRAs for those that are likely to be infected with Mtb [70]. In high-risk populations (TST ≥ 5 mm indicating likely to be infected and high risk of progression to ATB), both IGRA and TST are acceptable as screening tests for adults, while TST is preferable for children < 5 years old. In intermediate-risk populations (TST ≥ 10 mm indicating likely to be infected and low/intermediate-risk of progression to ATB), IGRA is preferable. In low-risk populations (TST ≥ 15 mm indicating unlikely to be infected), testing for TBI is not recommended. Repeated screening should be considered for positive results among low-risk patients and in the case of negative results among high-risk patients [70].

#### 2.3.1. Children 

Children < 5 years old are at higher risk of developing ATB and pose an increased risk of severe forms of the disease [71]. Despite the specificity of IGRA for BCG-vaccinated individuals, this test has not been entirely validated by the CDC for testing in children < 5 years old because fewer studies had been conducted previously, the difficulty in blood collection, and the high rate of indeterminate results [72,73]. However, other studies showed that IGRAs may not be influenced significantly by age <5 years [74]. Some other studies showed that IGRAs have similar sensitivity to TST in children <5 years although caution on the interpretation of both tests in children < 2 years old. [75,76]. While for children aged ≥5–18 years, IGRAs had greater sensitivity than TST. Interestingly, several studies have shown that none of the untreated children with negative IGRA progressed to ATB even with discordant test results TST^+^/IGRA^−^, suggesting that clinicians can rely on IGRA negative results as TST might be causing overdiagnosis and unnecessary treatment [77,78]. Although repeated IGRA testing was required for indeterminate results, the percentage is lower than those who fail to return for TST reads [77]. Both QFT^®^-GIT and T-SPOT^®^.TB are in high agreement (93%) for diagnosis of TBI in children, but moderate agreement (75%) was found between TST and QFT^®^-GIT; and TST and T-SPOT^®^.TB [79]. 

#### 2.3.2. Elderly

A study in adult populations showed that the results of both IGRAs were significantly age-dependent with a decreasing trend of sensitivity of 93.3%, 86.5%, 76.8%, and 68.3% for QFT^®^-GIT and 96.7%, 94.7%, 87.5%, and 85.7% in T-SPOT^®^.TB in the age groups of <29 years, 30–49 years, 50–69 years, and >70 years, respectively [80]. The sensitivity decline of T-SPOT^®^.TB according to age was not statistically significant when adjusted with factors such as absolute lymphocyte count, lymphopenia, C-reactive protein, being immunocompromised, location of TB lesion, and sex [80]. It is well known that lymphocyte counts and T cell-mediated immune responses decrease with increasing age. Nevertheless, the fixed number of cells (250,000 cells/well) that is used in T-SPOT^®^.TB may be the reason for the lesser decrease in sensitivity according to age compared to QFT^®^-GIT. Although a high number of discordant and inconclusive IGRA (indeterminate/borderline) results is observed in older people, a study performed in the Hispanic population showed that the combination of both QFT^®^-GIT and T-SPOT^®^.TB was suitable for TBI detection in older people [81].

#### 2.3.3. Immunocompromised Individuals

Immunocompromised patients who had low T cell counts or function, including recipients of lung and stem cell transplants, under immunosuppressive treatments or in conditions such as HIV/AIDS, diabetes, chronic renal failure, and cancer, are at a higher risk of developing ATB due to their weakened immune responses [82]. IGRAs show better sensitivity than TST in immunocompromised patients [83]. The fact of the fixed cell counts in T-SPOT^®^.TB might have also benefitted the use of the test for immunocompromised patients as it showed higher sensitivity (18.4%) compared to QFT^®^-GIT (15.1%) with lower indeterminate results of 0.6% and 7.2%, respectively [83]. Indeterminate QFT^®^-GIT results were associated with a lack of response in the mitogen tube, particularly in individuals under immunosuppressive treatments or with underlying conditions that affect their immune responses [55]. Following a positive diagnosis with T-SPOT^®^.TB, a higher rate of progression to ATB (10.37%) was observed compared to positive results with QFT^®^-GIT (0.65%) and TST (≥10 mm) (3.74%) [84]. The new version, QFT^®^-Plus, has shown improvement in the diagnosis of TBI among immunocompromised patients, with better concordance with T-SPOT^®^.TB (87.56%) compared to the older version, QFT^®^-GIT (83.19%) [85]. Despite the fact that IGRAs include a positive control tube, both tests have reduced sensitivity in detecting TBI [86,87]; therefore, IGRAs should be performed before initiating immunosuppressive treatment to rule out Mtb infection.

#### 2.3.4. BCG-Vaccinated

BCG is the oldest vaccine currently in use and one of the most widely administered vaccines worldwide [88,89]. Besides conferring protection against severe forms of TB deaths in children, BCG has also demonstrated nonspecific beneficial effects in preventing general infant morbidity and mortality [90,91,92]. In high TB endemic countries, the administration of the BCG vaccine is recommended for infants. This has greatly influenced the specificity of TSTs in the BCG-vaccinated population, leading to a higher number of false-positive results. In this regard, the use of IGRAs, which are less affected by BCG vaccination, could avoid unnecessary treatment for TBI [93,94]. False positive TST results derived from infant BCG vaccinations are frequent during the first two years after its administration, but may persist for decades [95,96].

#### 2.3.5. High-Endemic TB Countries

According to Dowdy and Behr, 2022, the current annual risk of TB infection, estimated around 1% in most high-burden countries, could be underestimated and could reach 5–10% because primary surveys on tuberculin were conducted in children aged 5–12 years, but the risk of infection is higher in those aged 15 years and older, some people may have false-negative results, and transient immune responses are not taken into account [97]. A survey on policies and tools implementation for TBI diagnosis and management in 24 high TB-burden countries showed that only five countries, i.e., Brazil, Lesotho, Mozambique, Russia, and Zambia, have full implementation of TBI guidelines, while no TBI guidelines were available in Angola, China, Democratic Republic of the Congo, India, Indonesia, Kenya, and Myanmar, mostly because the focus was on ATB management, not TBI management [98]. Three countries, i.e., China, Indonesia, and Russia, have their local manufacturer of PPD. Nine countries had experienced PPD shortages in the previous year, i.e., Brazil, India, Kenya, Pakistan, Philippines, South Africa, Thailand, Vietnam, and Zimbabwe [98]. In National TB Programs, only six countries, i.e., Cambodia, China, Nigeria, Russia, Tanzania, and Thailand, used IGRAs. The lack of budget allocation and facilities were the main problems associated with limitations in the use of IGRAs [98].

According to WHO, either TST or IGRA can be used as a screening test for TBI in high TB burden settings; however, TSTs may require fewer resources and may also be more familiar to TB practitioners [14].

Immigrants from high TB endemic countries are at risk of developing ATB within the first 2 years after migration [99]. IGRA is recommended to screen immigrants that move from TB endemic countries to low-incidence countries because fewer immigrants tested positive for IGRA and more IGRA-positive immigrants develop ATB compared to TST [100]. Additionally, the post-travel IGRA test is a useful screening tool for long-term travelers to high-endemic TB countries, at least 8 weeks after return [101]. Nonetheless, both TSTs or IGRAs may pose suboptimal sensitivities in newly arrived migrants, so TB symptoms screening should also be performed on arrival in order to detect ATB diseases [102].

#### 2.3.6. Healthcare Workers

Healthcare workers (HCWs) is at a high risk of acquiring TB through occupational exposure. A systematic review by Apriani et al. (2019) on the prevalence of TBI among HCWs in 26 low- and middle-income countries showed positive TST was 14–98% and positive IGRA was 9–86%, and the highest prevalence was in countries with a high TB incidence of ≥300 per 100,000. Positive test results among the HCWs are related to years of work, places of work, TB contact, and occupational category [103]. Occupational screening and periodic monitoring are recommended for HCWs who are at high risk of TB infection. Prevention of TB in HCWs relies on respiratory protection and administrative and environmental measures [104,105]. In addition, at least once a year screening is recommended for high-risk HCWs working in departments of pulmonology, TB infection clinics, respiratory and emergency departments, intensive care units, bronchoscopy suites, sputum induction rooms, spirometry rooms, TB-related laboratories, and aerosol-producing rooms [106].

In a study of HCWs working in TB-related departments in Korea (a country with a medium-burden TB incidence and high BCG coverage), high rates of TST conversion (baseline TST < 10 mm and follow-up TST ≥ 10 mm, with an increment of 6 mm from baseline in two years) were recorded; between 7–30%. Interestingly, only 26% of TST converters were IGRA-positive and none of the TST^+^/IGRA^−^ employers progressed to ATB during the follow-up period [107]. Another large study from USA HCWs showed that 66% of TST converters were IGRA-negative. Among 123 employers who converted TST, only 44% commenced TBI treatment and none developed ATB [108]. This study prompted CDC to change recommendations on serial testing in HCW, being now not routinely recommended unless identification of Mtb exposure or considering it only in certain groups with increased occupational risk exposure [109]. IGRAs, rather than TSTs, might be better selected for serial testing results given their 8-fold higher risk of progression to ATB in QFT converters compared to non-converters and their high negative predictive value for the risk of developing ATB [110]. Close observation of HCW is highly recommended for employers with initial borderline IGRA results, particularly for those HCWs in TB low-endemic countries due to the high rate of conversion and reversion [55,111].

## 3. Evaluation of Host-Derived Biomarkers

The current molecular assays for the detection of TBI are solely based on IFN-γ secretion after Mtb Ag stimulation. However, these tests have limitations such as the inability to differentiate between TBI and ATB, and are not entirely validated for their use in children < 5 years who are considered one of the high-risk populations [41]. In this section, we will discuss the use of other cytokines and chemokines that have been implicated in the pathogenesis and control of Mtb infection. 

In terms of the diagnostic platform, both ELISA (QFT^®^) and spot counts (T-SPOT^®^) could only measure a single analytic determination at a time and with a relatively significant volume of sample, which makes them unsuitable for multiplexing analysis. Flow cytometry has enabled the quantification of multiple cytokines in serum/plasma via multiplex immunoassay, which detects the fluorescent signals of Ab-coated microspheres. Additionally, it enables T-cell subset characterization, by lymphocyte immunophenotyping, allowing the simultaneous detection of cell markers and cytokines [112]. Another method providing automation and multiplexing is real-time quantitative PCR (RT-qPCR), which provides higher sensitivity than IGRAs for the detection of ATB and TBI [113].

### 3.1. Cytokines/Chemokines

Even at the baseline level, serum protein profiles showed that circulating cytokines (IFN-γ, TNF-α, IL-17A, and IL-17F) were significantly higher in PTB compared with both TBI and healthy control (HC) individuals [114]. Additionally, PTB individuals with bilateral or cavitary disease and high bacterial burdens had higher levels of IFN-γ, TNF-α, IL-17A, and IL-1β [114]. Similarly, another study had shown significantly higher IFN-γ and TNF-α in the serum of PTB compared to TBI [115]. The Mtb-specific immune response showed that multiple cytokines, i.e., IFN-γ, IL-2, IL-6, IL-8, IP-10, MCP-1, MIP-1β, and TNF-α, were released when blood from patients with TB (cultured-confirmed) is stimulated with Mtb Ags (ESAT-6+CFP-10+TB7.7) [49]. Our team also demonstrated that the cytokine levels after the stimulation with fusion proteins of these Ags in coated polyester nanobeads could be used to differentiate PTB, TST^+^, and TST^−^ healthy individuals by measuring the difference or ratio with unstimulated cytokine levels [116]. IL-2 and CCL11 were the best individual cytokines to differentiate between PTB and healthy individuals (TST^+^ and TST^−^) with a sensitivity and specificity of >80%, and the best combinations were IP-10+IL-2 and IFN-γ+IP-10+IL-2 with a sensitivity and specificity of >90%. These markers also had a similar sensitivity to differentiate between PTB and TBI (TST^+^) [116]. Immunological profiles with multiple markers showed that IFN-γ, IP-10, IL-2, IL-6, CCL3, and CCL8 could not only be used as biomarker signatures for TBI, but also to elucidate the risk of reactivation [117]. Additionally, the Mtb Ag-specific IFN-γ, TNF-α, and IL-2 levels could help in predicting successful anti-TB treatment and Mtb clearance [118].

Here, we will discuss the suitability of commonly reported cytokines/chemokines, including IL-2, IP-10, TNF-α, IL-10, and VEGF as adjunct markers for IGRA to aid in the diagnosis of TBI based on serum profiles at unstimulated and stimulated conditions, and a literature search, including meta-analysis studies. The determination of multiple cytokines upon stimulation with Mtb-specific Ags may help identify potential biomarkers for the diagnosis of TB and discriminate ATB from TBI, as shown in Table 3 [116,119,120,121,122,123,124,125,126,127]. 

#### 3.1.1. Interferon-γ (IFN-γ)

IFN-γ is a central effector of cell-mediated immunity with a critical role in recognizing and eliminating pathogens [128]. In mycobacterial infections, IFN-γ released by CD4^+^ T cells is essential for host survival and enhances both CD4^+^ and CD8^+^ T cell activities [129]. However, false-negative IGRA results were detected in patients with TB infection with advanced age [46,47,48,49,50,51,52,53,54,55,56,57,58,59,60,61,62,63,64,65,66,67,68,69,70,71,72,73,74,75,76,77,78,79,80,81,82,83,84,85,86,87,88,89,90,91,92,93,94,95,96,97,98,99,100,101,102,103,104,105,106,107,108,109,110,111,112,113,114,115,116,117,118,119,120,121,122,123,124,125,126,127,128,129,130,131,132], alcohol abuse [130], inflammatory diseases [130], HIV co-infection [46,132], non-Hispanic white race/ethnicity [46], longer time from diagnosis to treatment initiation [46], over-weight (BMI ≥ 25 kg/m^2^) [131], malnutrition (BMI <16 kg/m^2^) [132], longer period of illness before hospitalization [131], glucocorticoids and other immunosuppressive use [86,87,133], low peripherical lymphocyte counts [134] and extrapulmonary TB [133,135], among other factors. A study by Kellar et al. failed to show IFN-γ response in culture-confirmed TB patients, but other cytokines, i.e., IL-2, IL-6, IL-8, IP-10, and MIP-1β showed greater responses to ESAT-6, CFP-10, and TB7.7 Mtb Ags, suggesting that other cytokines or chemokines are potentially useful for the diagnosis of TB [49].

A meta-analysis on the diagnostic accuracy of IFN-γ for differentiating ATB from TBI showed an overall pooled sensitivity, specificity, negative likelihood rate (NLR), positive likelihood rate (PLR), diagnostic odds ratio (DOR), and area under curve (AUC) of 0.72, 0.82, 0.34, 4.0, 12.00, and 0.84, respectively [136].

#### 3.1.2. Interleukin-2 (IL-2) 

IL-2 is a cytokine with growth-promoting effects, promoting the differentiation of T cells into effector and memory T cells upon stimulation by an Ag, thus helping the body to fight off infections [137]. In a study among household contacts of pulmonary TB patients, the ESAT-6/CFP-10-stimulated IL-2 level was significantly higher among TB-infected compared to non-TB-infected subjects as a standalone marker, but it did not discriminate between ATB and TBI [138]. A prospective study conducted in China showed that the combination of IFN-γ and IL-2 in the supernatant of stimulated Mtb antigens may differentiate between active TB and TBI [139]. A study that evaluated forty-eight cytokines, chemokines and growth factors showed that IL-2 was the biomarker most frequently included among the top seven biomarkers to detect Mtb-infected individuals, followed by IFN-γ [126]. A recent study that evaluated cytokines/chemokines other than IFN-ɣ in the supernatants of Quantiferon^®^-TB showed that IL-2 along with RANTES Ag could be used as an alternative to differentiate ATB from TBI [140]. Nonetheless, some other studies failed to find IL-2 as a useful biomarker that can distinguish ATB from TBI [141].

The sensitivity of IL-2 might be influenced by the Ag stimulation time as shown by Biselli et al. (2010), IL-2 response in QFT^®^-GIT after 18 h of incubation was low and not significant, but prolonged incubation of 72 h significantly increased the response in TBI (median: 14.72 U/mL) compared to ATB (0.44 U/mL) and HC (0.11 U/mL), with AUC 0.99 [142]. Ag-stimulated IL-2 and IFN-γ after 72 h were significantly different between ATB and TBI, where the IL-2 response was higher in TBI, and IFN-γ was higher in ATB [143]. The IL-2/IFN-γ ratio had an AUC of 0.9504 and 0.8916 after the stimulation with PPD and ESAT-6/CFP-10, respectively [143]. Using flow cytometric cytokine-secreting cell detection, after 72 h of PPD incubation, IL-2 secreting cells were more frequently observed in TBI than in ATB compared to stimulation with ESAT-6/CFP-10 [144]. Another study showed that, although the coefficient correlation of the IL-2/IFN-γ ratio (0.77) is higher than the TNF-α/IL-2 ratio (0.74), the latter showed better discrimination between ATB and TBI [145]. Another study that suggested subtracting IFN-γ with IL-2 showed a higher AUC of 0.8910 in patients with ATB rather than comparing their ratio (0.7164) [123].

A meta-analysis of multiple cytokines showed that IL-2 had the highest accuracy to assist distinction between TBI and ATB, followed by IP-10, IL-5, IL-13, IFN-γ, IL-10, and TNF-α [146]. A meta-analysis on the diagnostic accuracy of IL-2 for differentiating active TB from TBI showed an overall pooled sensitivity, specificity, NLR, PLR, DOR, and AUC of 0.83–0.84, 0.66–0.76, 0.22–0.24, 3.41–2.5, 10.00–15.47, and 0.84–0.87, respectively [136,147].

#### 3.1.3. IFN-γ-Inducible Protein 10 kDa (IP-10)

IP-10 also known as CXCL10, is a pro-inflammatory chemokine [148]. IP-10 levels were higher than IFN-γ levels in ATB and TBI patients following Mtb Ag stimulation (ESAT-6, CFP-10, and/or TB7.7) [149,150]. IP-10 increased significantly in ATB and TBI compared to HC, but could not discriminate ATB from TBI in both adult and children populations [149,150,151]. The measurement of IP-10 in saliva samples proved its value for discriminating ATB patients from HC and TBI [152]. Unlike IFN-γ, the expression of IP-10 was stable, not age-dependent, and was able to identify more positive results in children aged <5 years who had the risk of exposure to TB infection [153]. Thus, it was suggested as a potential adjunct marker, in combination with IFN-γ in IGRAs for screening in children aged <5 years [153]. 

Another study using RNA from stimulated cells in QFT^®^-GIT showed that IP-10 mRNA levels had significant increases in children aged <18 years old with ATB and TBI than in HC [113]. Additionally, the AUC of IP-10 mRNA level was higher (0.78) than IFN-γ (0.59) in discriminating between ATB and TBI [113]. However, the IP-10 mRNA had a lower sensitivity (80%) compared to the protein-based IP-10 release assay (87%) due to the different mRNA expression kinetics between patients [154], thus optimization of stimulation times before RNA extraction is highly recommended.

Besides high sensitivity in the diagnosis of TB, the use of an IP-10 marker could be the solution for indeterminate results in QFT. An analysis of cytokines in QFT^®^-GIT supernatants showed that IP-10 had the highest AUC (0.92) in differentiating QFT borderline and QFT negative controls, followed by IL-2 (0.875) and IL-1ra (0.852) [155]. These findings were confirmed in a recent study by Uzorka et al., which found that levels of IP-10 and Monokine Induced by IFN-ɣ (MIG) in supernatants of QFT predicted true TBI among patients with borderline IFN-ɣ levels in QFT^®^-Plus [156].

A meta-analysis on the diagnostic accuracy of IP-10 for differentiating active TB from TBI showed an overall pooled sensitivity, specificity, NLR, PLR, DOR, and AUC of 0.72–0.86, 0.83–0.89, 0.16–0.32, 4.63–7.55, 17.86–44.23, and 0.8638–0.93, respectively [157,158,159]. IP-10 may also correlate with treatment response in ATB patients [160].

#### 3.1.4. Tumor Necrosis Factor-α (TNF-α) 

TNF-α is a proinflammatory cytokine secreted by macrophages in response to cell damage caused by infection and regulates cell functions including proliferation, survival, differentiation, and apoptosis [161]. Secretion of TNF-α by ESAT-6 or CFP-10-stimulated PBMCs was significantly increased in the ATB group compared to HC, but TNF-α levels do not distinguish ATB from TBI or non-TB controls due to the high background of TNF-α under unstimulated conditions [162]. By subtracting the background levels, the AUC showed a significant difference in TNF-α levels in discriminating between ATB and TBI under CFP-10 stimulation (0.94) compared to ESAT-6 stimulation (0.72) [162]. A similar result was observed by Harari et al. (2011) reporting that CFP-10-stimulated TNF-α positive cells were more frequently recognized than ESAT-6-stimulated cells via flow cytometry assay [163]. The proportion of TNF-α single-positive Mtb-specific CD4+ T cells was the strongest predictive measure of discrimination between ATB and TBI with an AUC of 0.995 [163]. Contradictory results were observed by Zhang et al. (2021), in which ESAT-6 was a better stimulant than CFP-10 for the detection of IFN-γ and TNF-α by a fluorospot assay [164]. The AUC of ESAT-6-stimulated TNF-α (0.970) was higher than IFN-γ (0.872) in discriminating between ATB from TBI. Although a combination of IFN-γ and TNF-α levels had lower AUC (0.900), the specificity of the test increased to 97.1% from single TNF-α (94.3%) and IFN-γ (77.1%) [164]. A study by Kim et al. (2020) had also shown that the diagnostic specificity of the ESAT-6 and CFP-10-stimulated in an IFN-γ/TNF-α dual release assay (94%) by fluorospot was greater than the IFN-γ single release assay alone (72%) [165].

RNA from whole blood cells under Mtb-specific Ag stimulation in QFT-GIT showed that the TNF-α mRNA level had a statistically significant increase in ATB compared to TBI, and the combination of IFN-γ, TNF-α, and IL-2 receptor (IL-2R) showed the best performance in detecting ATB (100%) and TBI (86.36%) with an AUC of 0.9852 [166].

Another study suggested that the ratio of IFN-γ/TNF-α in response to either Rv3716c or TrxC may act as a suitable surrogate biomarker for TBI with an AUC of 0.96 [167].

A meta-analysis on the diagnostic accuracy of TNF-α for differentiating ATB from TBI showed an overall pooled sensitivity, specificity, NLR, PLR, DOR, and AUC of 0.70, 0.79, 0.37, 3.4, 9.00, and 0.81, respectively [136].

#### 3.1.5. Interleukin-10 (IL-10)

IL-10 is an anti-inflammatory cytokine, ameliorating the excessive Th1 and CD8^+^ T cell responses (typified by overproduction of IFN-γ and TNF-α) that are responsible for much of the immunopathology associated with infections [168]. IL-10 mRNA expression levels in PBMCs after ESAT-6 and CFP-10 antigenic stimulation correlates with negative IGRA results in culture-confirmed TB patients [133]. Another study suggested that the combination of IL-6 and IL-10 with QFT and/or TST could markedly improve the detection accuracy of TBI as IL-6 had the highest positivity rate (92.59%) in the QFT^+^/TST^+^ group, and IL-10 had the highest positivity in the QFT^−^/TST^−^ group [169]. 

#### 3.1.6. Vascular Endothelial Growth Factors (VEGF) 

VEGFs are central regulators of angiogenesis and lymphangiogenesis [170]. VEGFs have been shown to be good markers for discriminating TBI and ATB even at the baseline level [118]. Serum IL-22, IP-10, and VEGF-A were significantly higher in ATB patients than in HC, while only VEGF-A could differentiate between ATB and TBI with an AUC of 0.7576 [118]. Serum CCL1, IP-10, VEGF, and ADA_2_ significantly increased in ATB than TBI and in combination were able to differentiate between the two groups with an AUC of 0.9525, sensitivity of 95%, and specificity of 90% [171]. Serum VEGF-A and VEGF-R2 levels were significantly higher in PTB compared to TBI individuals with an AUC of 0.9933 and 0.9995, respectively [172].

A meta-analysis on the diagnostic accuracy of VEGF for differentiating ATB from TBI showed an overall pooled sensitivity, specificity, NLR, PLR, DOR, and AUC of 0.59, 0.87, 0.47, 4.5, 10.00, and 0.85, respectively, and it had the highest AUC to assist distinction between TBI and active TB, followed by IFN-γ and IL-2 [136].

### 3.2. mRNAs and microRNAs

Advancements in next-generation sequencing have enabled the use of transcriptomic profiling to understand the transcriptome dynamics and the discovery of new biomarkers related to a disease [173]. The mRNAs, which are related to various key biological processes including immune defense, inflammatory responses, cell activation, cell proliferation, and apoptosis, among others, could be used as genetic signatures indicative of ATB and TBI. These gene expressions are regulated by microRNAs (miRNAs) post-transcriptionally [173]. miRNAs are host short non-coding RNAs that interact with complementary mRNAs, resulting in positive regulation (transcription stimulation) or negative regulation (transcription inhibition or mRNA degradation) [173]. As shown in Table 4, multiple miRNAs could also serve as biomarkers for ATB and TBI [174,175,176,177,178], but are probably not yet ready for everyday use in TB clinics [179]. In addition to miRNAs, one study showed that small nuclear RNA (snoRNA) and PIWI-interacting RNA (piRNA) are important biomarkers for TBI with potential participation in the TB pathophysiology [180]. 

A study by Zak et al. (2016) had identified 16 gene signatures of risk that could predict TB progression by whole blood RNA sequencing of adolescents (12–18 years) with latent Mtb infection who developed ATB in a 2-year follow-up [181]. Further study on these genes revealed that 57 primer-probes for 16 genes and 48 primer-probes for 11 genes (RISK11) had similar diagnostic performances [182]. The RISK11 could also be used to discriminate between adults (18–59 years) with prevalent TB and those who remained healthy [183]. Individuals with both positive RISK11 and QFT^®^-Plus tests had an 8.3-fold increased risk of incident TB than individuals with both tests negative [184]. RNAseq analysis by machine-learning identified patients with TBI with an ATB profile, suggesting Mtb infection with a high risk of progression to ATB [185].

### 3.3. T-Cell Subsets 

Flow-cytometry-based assays have enabled the detection of a broad population of Ag-specific T cells from Th1, Th2, Th17, Tfh, and Treg lineages [186]. Studies on the cell surface marker expression, also known as immunophenotyping, enable the identification of TB-specific immune phenotypes, which can be used for the diagnosis of the disease, to distinguish ATB from TBI, and to determine the risk of developing TB [187].

TB patients (extrapulmonary TB (EPTB) and PTB) had higher frequencies of ESAT-6/CFP-10–specific IFN-γ CD4 T-cells expressing CD38, HLA-DR, or Ki67 compared with TBI, while EPTB had higher frequencies of cells expressing HLA-DR or Ki67 compared with PTB [188]. Additionally, HLA-DR (an MHC class II member) expression by ESAT-6/CFP-10-specific CD4 T cells had the highest diagnostic performance to distinguish between recent and remote TB infections [189].

CD4 T cells co-expressing the surface marker CD25 (subunit of IL-2 receptor) and CD134 (OX40, a TNF-α receptor superfamily member) after ESAT-6 and CFP-10 stimulation had high diagnostic accuracy for ATB and HIV infection [190]. Additionally, patients with IGRA^+^ CD4^+^ CD25^+^ CD134^+^ T cell phenotypes had highest estimated TB reactivation risk [191].

In a study from Spain, ATB patients presented a higher monocyte to lymphocyte ratio than TBI and HC. In the same study, ATB patients showed a lower proportion of Central Memory cells (T_CM_) or Mucosal-associated invariant T cells (MAIT) than TBI and HC. In addition, CD-154 expression was increased in T_CM_ and effector memory T cells in patients with ATB, suggesting a potential role in distinguishing ATB from TBI and HC [192].

### 3.4. Gene Polymorphisms

Whole-genome sequencing technologies have enabled the mining of potential genes associated with susceptibility to TB. Using the Sure Select kit, 1611 single nucleotide polymorphisms (SNPs) were identified in ATB but not TBI, while the TruSight kit identified 182 single-nucleotide polymorphisms (SNPs) [193]. SNPs related to ATB but not with TBI were found to belong to Toll-like receptor-1 (TLR1), vitamin D receptor (VDR), and tumor necrosis factor (TNF) [193].

Gene polymorphism has been associated with PTB and TBI. In a study on Toll-like receptor (*TLR*) gene single nucleotide polymorphisms (SNPs) in the Chinese population, CC genotype and C allele in SNP rs3804100 (*TLR2*) and TC genotype in SNP rs5743836 (*TLR9*) were significantly more common in TBI than in HC. GA genotype and G allele in SNP rs5743708 (*TLR2*), T allele in SNP rs4986791 (*TLR4*), GG genotype in SNP rs7873784 (*TLR4*), CC genotype in SNP rs3764879 (*TLR8*), and T allele in SNP rs8177374 (toll-interleukin 1 receptor (TIR) domain containing adaptor protein (*TIRAP*)) were significantly more common in PTB than in HC [194]. There were no significant differences in genotype or allele frequencies between PTB and TBI using single genetic markers, but a combination of a three-markers from *TLR4* (rs10759932, rs7873784, and rs10759931) had a predicted accuracy of 84% for TBI [194]. 

In the Chinese population, the CC+CT genotype in rs1861494 of *IFN-γ* had decreased the risk of TBI by 50%, while A allele in rs2234711 of *IFN-γ* receptor 1 had increased the risk of TBI by 55% [195]. An allele at nucleotide -874 of *IFN-γ* was significantly common in both PTB and TBI compared to HC, and A allele at nucleotide −1082 of *IL-10* was significantly more common in PTB patients than in TBI subjects [196].

A study in Taiwan showed that SNPs in the *SP110* gene (encoding an interferon-induced nuclear protein) were associated with susceptibility to TB. In TBI vs. HC cases, GG genotype in rs7580912 and GG genotype in rs7580900 were associated with TBI risk, while GA genotype in rs9061 exhibited a protective effect on TBI. Additionally, a protective effect on TB was observed in GG genotype in rs7580912, GG genotype in rs7580900, and CT genotype in rs11556887. It was also observed that the GA genotype of rs9061 in TBI individuals was associated with lower TNF-α levels in plasma compared to GG genotype [197].

In the Mexican population, the G allele and the GG genotype of rs1135216 of transporter associated with Ag processing (*TAP1*) were associated with susceptibility to TBI [198].

In the Brazilian population, C allele in rs1101998 and A allele in rs1633256 of *PYHIN1- IFI16-AIM2* were associated with an increased risk of TST-positivity among close contacts [199].

A recent study performed in the Chinese population demonstrated SNPs in Transforming Growth Factors (TGF)-β1 genes were associated with increased susceptibility to TB and severe forms of the disease [200]. 

### 3.5. Host Circulating Proteins and Metabolites

Proteomic analysis of plasma from ATB, TBI, NTM, and HC patients with and without ESAT-6/CFP-10 stimulation, using liquid chromatography-mass spectrometry (LC-MS/MS) showed an increase of the enzyme M7GpppN-mRNA hydrolase (DCP2) only in the TBI group, while C-reactive protein (CRP), α-1-acid glycoprotein 1 (ORM1), sialic acid-binding Ig-like lectin 16 (SIGLEC-16), and vitamin K-dependent protein S (PROS1) increased in abundance in ATB compared to TBI [201].

Proteome urine analysis of urinary samples from ATB and non-TB controls showed that 902 peptides and 160 proteins were unique to ATB patients. Selected targets were validated using multiple reaction monitoring (MRM), and ROC analysis showed that a combination of five biomarkers, i.e., P22352 (glutathione peroxidase 3), Q9P121 (neurotrimin), P15151 (poliovirus receptor), Q13291 (signaling lymphocytic activation molecule family 1), and Q8NDA2 (hemicentin-2), had the best accuracy in the diagnosis of ATB. Out of these five panels, a three-protein combination (Q9P121, P15151, and Q8NDA2) showed a sensitivity rate of 82.7% in the diagnosis of ATB from non-TB and a specificity of 92.3% for the diagnosis of ATB from the TBI group [202].

Metabolomic analysis of urine from ATB, TBI, and HC using ultrahigh-performance liquid chromatography-tandem hybrid quadrupole-Exactive Orbitrap mass spectrometry (UPLC-Q Exactive MS) showed that glutathione (GSH) and histamine could be used as potential markers to differentiate between TBI vs. HC, ATB vs. HC, and ATB vs. TBI with an AUC of 0.763, 0.982, and 0.880 for GSH and 0.926, 0.998, and 0.884 for histamine, respectively. Quantitative analysis using ELISA showed that the levels of GSH and histamine were highest in non-infected individuals, followed by TBI, and lowest in ATB [203].

A proteomic study of saliva and sputum performed using an LTQ-Orbitrap-Elite platform in Spanish patients with ATB and their contacts found specific proteomic signatures involved in the perception of bitter taste, defense against pathogens and innate immune response. The results obtained in the study were suggestive that those signatures are key events during the initial entry of the Mtb in the host [204]. Another study performed in the same population and a validation cohort in Mozambican patients demonstrated decreased specific protein signatures related to lipid transport and iron assimilation in ATB patients, suggesting their importance in the immune control of the disease [205].

## 4. Evaluation of Mtb-Derived Biomarkers

Stimulating Ags add value to immune profiling and may enhance the IGRA sensitivity. As shown by Robinson et al. (2021), the use of MTB300, 300 Mtb-derived T cell epitopes that specifically target a large fraction of Mtb-specific CD4^+^ and CD8^+^ T cells improved the diagnostic accuracy of TBI [117]. Hence, in this section, we will review the Mtb-specific Ags that could serve as an alternative or complementary stimulating Ag to ESAT-6 and CFP-10.

### 4.1. Mtb Latency Antigens

Genome-wide transcriptome profiling had identified protein-coding genes upregulated during TBI in in vitro models of latency known as “latency Ags” [45]. Some of the proteins involved in the reactivation of the dormant bacteria were released in TBI patients, i.e., dormancy survival regulon (DosR regulon) Ags, resuscitation-promoting factors (Rpf) Ags, in vivo-expressed Mtb (IVE-TB) Ags, and reactivation associated Ags [206]. A study showed that the T cell response to these latency Ags was significantly higher in TBI than in ATB [207]. 

Among 25 latency Ags tested by Leyten et al. (2006), Rv1733c, Rv2029c, Rv2627c, and Rv2628 induced strong IFN-γ responses in 61%, 61%, 52%, and 35% of TST^+^ individuals, respectively [207]. A study by Serra-Vidal et al. (2014) on 60 latency-related Ags also showed that Rv1773 was the most immunogenic protein that can distinguish between non-infected, TBI, and ATB patients based on the IFN-γ response after 18 h of stimulation, with higher responses in TBI [206]. Another study on stimulation with synthetic long peptides derived from Rv1773c Ag (Rv1773c SLP), IL-2 secreting T-cells were significantly higher in TBI compared to ATB, but no significant results were obtained with IFN-γ secreting T-cells [208].

PBMCs stimulated with the DosR Ags, Rv1737c, Rv2029c, and Rv2628 showed that TBI had higher IFN-γ levels compared to ATB with an AUC of 0.76, 0.82, and 0.72, respectively [209]. In remote TBI (≥3 years since infection) IFN-γ response to Rv2628 for short (1-day) and long (7-day) incubation intervals were significantly higher than in recently infected individuals (≤3 months since infection), but no significant difference was found after stimulation with Rv2626c, Rv2627c, Rv2031c, and Rv2032. A higher proportion of QFT^+^ TBI (87.5%) had IFN-γ response to Rv2628 compared to QFT^+^ ATB (24%) with an AUC of 0.85 [210].

In a study by Adankwah et al. (2021), a combination of Rv1733 and Rv2628, induced high cytokines response in asymptomatic contacts compared to TB patients, particularly IL-6 [211].

Among Rv2624c, Rv2626c, and Rv2628, only the Rv2626c-stimulated IFN-γ response was significantly higher in TBI compared to BCG-vaccinated healthy donors [212]. Additionally, Rv2626c allowed the discrimination between ATB and TBI with an AUC of 0.8579, suggesting that this Ag could improve TBI diagnosis even in the BCG-vaccinated population [212]. Another study showed that 43% of healthcare workers that were exposed to Mtb for more than two years who were QFT^−^ had significant IFN-γ secretion against Rv2626c [213]. However, 69% of close contacts who were exposed to Mtb for less than 3 months with QFT^+^ did not respond to Rv2626c stimulation [213]. Overall, these data suggested that Rv2626c would reduce false QFT^−^ results in individuals that have long-term exposure to Mtb, and could be used to discriminate between latent Mtb and recent infection [213]. A similar pattern of IFN-γ and IgG anti-Rv2626c plasma levels was observed [213]. Rv2626c-stimulated IFN-γ exhibited greater discrimination between PTB and household contacts than TNF-α [213]. A combination of Rv2626c and Rv3716c showed 100% positivity in household contacts and 17.5% in PTB, thus, improving the TBI detection rate [213]. 

Rv2031 (also known as Hsp16.3, Hsp16, 16 kDa, HspX, or α-crystalline (Acr)) is one of the most immunogenic DosR Ags, associated with the long-term viability of Mtb during the dormancy period [214]. IFN-γ, TNF-α, and IL-10 responses to Rv2031 were significantly higher in healthy controls compared to contacts and untreated TB patients at baseline [215]. After 12 months, these cytokine responses increased in contacts and treated TB patients with comparable levels to controls, suggesting that Rv2031 could be used as a protective marker for TB [215]. Another study also demonstrated that the IFN-γ response to Rv2031 correlated with protection against TB and the IFN-γ ratio of responses to ESAT-6 and Rv2031 could determine the risk of progressive or latent TB [216].

Other DosR Ags, e.g., Rv2004c [217], Rpf Ags, e.g., Rv0867c and Rv2389c [206,209,218], and IVE-TB Ags, e.g., Rv2435n [206], have shown significantly higher IFN-γ response in TBI compared to ATB.

### 4.2. Mtb Antigens Used for Serodiagnostic

Serological tests, i.e., Anda Biologicals TB IgG test (Anda-TB) (Ag 60), Pathozyme-Myco IgG test (Myco G) (LAM and 38 kDa (Rv0934)), IBL Mtb IgG ELISA test (18, 36, and 40 kDa), and Pathozyme TB Complex Plus test (TB complex) (38 and 16 kDa (Rv2031c)) showed AUC of 0.8309, 0.7336, 0.7110, and 0.7008, respectively, in discriminating TBI and ATB [219]. Despite the ease of using antibody detection tests, WHO warns against the use of serological tests for the diagnosis of ATB due to their inaccurate/unreliable results [220].

A serum profile analysis of TBI, ATB, and HC using a microarray containing 257 Mtb secreted proteins identified higher levels of Abs in ATB than TBI and HC against 23 Mtb Ags. Four of the Ags, i.e., Rv0934 (38 kDa), Rv1860, Rv3881c, and Rv1827 showed a significant difference between ATB vs. TBI and ATB vs. HC [221].

Humoral responses to DosR Ag and Rv2031 (Hsp16) were minimal in ATB, but Mtb latently infected individuals who were chronically exposed to Mtb had high titers of anti-Acr IgA Abs against this Ag [222]. Another study also showed that anti-HspX IgG and IgM Abs in recent TBI (<1 year since infection) were significantly higher than in ATB, previous TBI, and uninfected individuals [223]. Ab responses against Mtb Hsp16 Ag were significantly higher in TBI compared to Hsp65 and Hsp71 [224]. 

In a study of Ab responses to 15 Mtb Ags, only Rv1733c was recognized by IgG from endemic controls compared to TB patients and non-endemic controls, suggesting its potential in controlling TB infection and progression [225].

Ab response against a combination of latency proteins Rv2029c, Rv2031c, Rv2032, Rv2627c, Rv3133c, and Rv3716c was able to diagnose TBI with a sensitivity of 75% [226]. 

The evaluation of the Ab response against Mtb-secreted proteins in ATB and TBI showed that IgG Ab responses to Ag combinations, such as Ag85B-Hsp16.3/ESAT6 and Hsp16.3/ESAT6, were the best markers for the diagnosis of ATB and TBI with a sensitivity and specificity of 92.39% and 93.33%; and 75.00% and 76.67%, respectively [227].

IgG and IgA Ab responses to Ag85B and IgG Ab responses to CFP-10 were significantly higher in ATB, followed by TBI, and lowest in non-infected subjects. ROC analysis showed that IgG against Ag85B was the most significant marker to diagnose and discriminate ATB and TBI with AUC of 0.9885 [228].

Recent TBI (<2 years since infection) had a higher risk of TB progression than remote TBI (>2 years since infection). IgG Ab titers against ESAT-6 and mycobacterial DNA-binding protein 1 (MDP1) were significantly higher in individuals with recent TBI than in those with no Mtb infection or remote TBI [229].

### 4.3. Detection of Mtb DNA in TBI

Broken bacterial gene fragments are believed to be released into the blood in the form of circulating cell-free DNA (cfDNA) [230]. A study showed that the plasma MTB-cfDNA might be useful as a microbiological indicator for Mtb infection in TBI. Using qPCR and Mtb-specific *IS*6110-cfDNA, the authors found high specificity for TBI (86.5%), but low sensitivity (two out of 57 TBI patients (3.5%)). The authors believed that these two patients may have an incipient TB stage, and they did not develop TB as they received prophylactic treatment for TBI. Additionally, it was observed that their *IS*6110-cfDNA levels declined after treatment [230].

In a study of 100 TBI patients (TST ≥ 10 mm) with rhegmatogenous retinal detachment undergoing pars plana vitreous surgery with internal tamponade, PCR analysis in retinal pigment epithelium cells to detect Mtb by targeting three genes, i.e., *IS*6110, MPB64, and protein b, showed that three samples were positive with all the three genes and three samples were positive with *IS*6110 and MPB64 [231].

CD271^+^ and CD34^+^ are bone marrow stem cells that serve as potential hosts for dormant Mtb. A higher copy number of Mtb DNA was detected in CD271^+^ cells compared to CD271^−^ cells [232]. Additionally, viable Mtb was detected in CD271^+^ cells after the completion of antitubercular treatment, suggesting CD271^+^ as a reservoir for dormant non-replicating Mtb and a possible source of reinfection in the host [232]. MTBC DNA was more commonly detected in CD34^+^ (73%) than CD34^−^ (23%) PBMCs [233].

### 4.4. Detection of Mtb Antigens in TBI

Proteomic analysis of urine from TB, TBI, and non-TB/non-TBI groups using mass-spectrometry showed the presence of five Mtb-specific proteins (i.e., PE-PGRS family proteins (Rv2126c and Rv 3345c), WAG22 Ag (Rv1759c), ATP-dependent DNA helicase (Rv3202c), and DNA-directed RNA polymerase subunit (Rv0668)) and one *Mycobacterium*-related protein (i.e., probable cysteine desulfurase (Rv1464)) were found exclusively in TBI but not in TB [234]. Four proteins were found in the urine of both TB and TBI, i.e., PE-PGRS family protein (Rv1450c), putative membrane protein mmpL12 (Rv1522c), RecA (Rv2737c), and D-alanine-D-alanine ligase (Rv2981c) [234].

A study with Luminex xMAP®/Magpix (Luminex corp, Austin, TX) bead-capture ELISA system showed that the median for HspX protein in serum detection was 860 pg/mL for TBI, 40 pg/mL for ATB, and 470 pg/mL for HC with 56.5% of TBI and 0% of ATB scoring above the median of HC [223].

Kim et al. (2020) developed a highly sensitive naked-eye detection of Mtb Ag85B in urine specimens using gold and copper nanoshell-enhanced immunoblotting techniques [235]. A higher signal intensity for Ag85B was observed in ATB urine specimens with an accuracy of 90.5%, and the Ag was detected in 62.5% of TBI patients. Although the Ag was not detected in HC individuals, it showed cross-reactivity in urine samples of non-tuberculous mycobacterial lung disease patients (33.3%) [235].

Mtb-secreted protein, ornithine carboamyltransferase (MT1694; Rv1656 [argF]) was discovered in the urine of PTB using mass spectrometry. The recombinant protein (rMT1694) was recognized by IgG Abs from ATB, but not from HC. Additionally, rMT1694 was recognized by PBMCs from ATB and PPD^+^ (TBI) individuals. An Ag detection assay was developed to detect the MT1694 in urine, and the results showed the presence of this Ag in 6 out of 16 ATB samples and none in PPD^+^ samples, suggesting this might be a potential marker for the development of a diagnostic test for TB and to distinguish it from TBI [236].

Mehaffy et al. (2017) developed a multiple reaction monitoring mass spectrometry (MRM-MS) assay for enhanced detection of ultra-low abundance Mtb peptides (41 peptides from 19 Mtb proteins) in exomes purified from the serum of TB patients [237]. In 2020, this method was used for the analysis of serum extracellular vesicles in TBI patients, and at least one Mtb peptide was detected in 95% of TBI samples. Peptide SVF from GlnA1 (Rv2220) was most commonly identified (82%), followed by peptide DVL from GroES (Rv3418c) (23%), TTP from DnaK (Rv0350) (19%), FLL from GarA (Rv1827) (16%), and IPD from AcpM (Rv2244) (16%) [238].

## 5. Clinical and Epidemiological Scoring 

Clinical and epidemiological scorings are important for accurate diagnosis of TBI as there is no gold standard test for TBI. Diagnosis is challenging for those persistently TST^−^/IGRA^−^ although they had high exposure risk with Mtb. Additionally, medical evaluations are necessary for TST^+^/IGRA^+^ individuals to rule out ATB. The decision on treatment for TBI will be based on a person’s risk factors for progression to ATB [239].

The BCG world atlas (http://www.bcgatlas.org/, accessed on 1 October 2022) is an interactive map that provides detailed information on current and past BCG vaccination policies and practices in over 200 countries. It also includes current TB incidence rates. These data are important while interpreting TST results and in deciding if an alternative test, such as IGRA, is preferable to TST. 

The online TST/IGRA interpreter, Version 3.0 (http://www.tstin3d.com/, accessed on 1 October 2022 ), is a three-dimensional tool that estimates the positive predictive value and risk of active TB based on TST size and/or IGRA results. The system takes into account the country of birth, age, age at immigration, BCG status, recent TB contacts, and other comorbidities. This algorithm is suitable for subjects aged 5 and above. It is recommended to start treatment if the estimated cumulative risk of active TB at the age of 80 is high and the drug toxicity risk is low. However, the calculator needs to update data on the country-specific prevalence of TB, as the global TB burden continues to decrease. Additionally, it does not take into account the quantitative results of IGRA, as it only categorizes them in a dichotomic way (i.e., “positive” or “negative”). Furthermore, falsely high positive predictive values could be produced by the system due to “transiently” positive IGRA results in subjects from low-prevalence countries [240]. 

A TB contact score was developed to evaluate the intensity of exposure. It was weighted based on the relationship to the TB index case, infectivity of the index case, proximity of exposure, and duration of exposure, with a score of <4 indicating low exposure and ≥4 indicating high exposure [241]. Another study performed in household contacts of TB patients in Peru developed two different tools that could help to predict the risk of TB during the first year after exposure to Mtb. The score is easy to perform as it is based solely on clinical information of the index patient and the contact [242].

For healthcare workers, a study recommended the use of clinical risk scoring based on the frequency of TB contacts inside hospital (≥6 times), working duration in the hospital (≥60 months), and age (≥30 years); and a cut-off of three points and greater for diagnosis of TBI [243].

## 6. Treatment for TBI

Treatment of TBI is essential for TB control because it reduces the risk of progression to ATB [244]. Individuals who receive treatment for TBI are not sick, so the decision to treat individuals with TBI and the type of treatment must carefully balance the risk of reactivation, the safety of the treatment, and the benefits to the individual. According to WHO, the individual benefit outweighing the risk should be the mainstay of TBI testing and treatment [244]. The factors that influence the decision to implement TBI treatment should be based on the expected change in cumulative quality-adjusted life-years (QALYs) such as underlying risks of developing TB, utility (value relative to perfect health) assigned to the post-TB state, utility assigned to uncomplicated treatment of TBI, and the effectiveness of treatment in reducing the risk of TBI developing into ATB [245]. The WHO guidelines recommend identifying TBI and treating patients living with HIV, household contacts, people who are initiating anti-TNF medication, receiving dialysis, and patients with silicosis. In addition, testing and treatment may be considered for prisoners, people who use drugs, homeless, and immigrants proceeding from a high-prevalence country [239].

### 6.1. Standard TBI Therapy

The CDC recommended the following prophylactic regimens for TBI, i.e., 6-month daily/twice weekly isoniazid (INH) (180/52 doses) (6H), 9-month daily/twice weekly INH (270/76 doses) (9H), 3-month daily INH + rifampicin (RIF) (90 doses) (3HR), 4-month daily RIF (120 doses) (4R), and 3-month once-weekly INH + rifapentine (RPT) (12 doses) (3HP) [246]. In people living with HIV, one month of daily INH + PPT (1HP) has also demonstrated efficacy in preventing ATB and higher rates of adherence to treatment [247,248].

The long duration of treatment and adverse drug reactions during TBI therapy are the most common barriers that lead to poor compliance. The completion of TBI therapy is related to short regimens, directly observed treatment, and treatments conducted in the frame of established TB control programs [249,250]. Non-adherence factors are patient-specific, personalized interventions such as mobile phone texting can address the issue of forgetfulness to take medications and lack of social support [251]. 

A study by Sterling et al., 2011, showed that the rate of completion of TBI treatment was higher with the 3HP regimen (82.1%) than with 9H (69.0%). Drug-related hepatoxicity was 0.4% and 2.7%, respectively [252]. Most of the TBI patients under the 9H regimen discontinued their treatment in the first 3 months due to severe or moderate hepatoxicity [253]. Although the 3HP regimen is not associated with hepatoxicity, it causes higher rates of adverse events such as fatigue, fever, and vomiting [254]. Nevertheless, these adverse events were mild and 3HP had high effectiveness in preventing TB, in which only 0.19% of the 3HP-treated group developed TB compared to 0.24% in the 9H group [252]. Additionally, 3HP is a cost-effective treatment compared to 9H, saving $1421 per 100 people tested [255]. Due to the safety and effectiveness of 3HP, CDC recommends this treatment for (1) TBI in adults, (2) TBI aged 2–17 years, and (3) TBI in people living with HIV with AIDS and under antiretroviral medications with acceptable drug–drug interactions with RPT [256]. Generally, CDC recommends short-course rifamycin-based regimens, 3HP, 3HR, and 4R, to treat TBI, and alternatively, the use of 6H and 9H if short-course treatment is not feasible or not available. Drug prescription should be modified if the index TB patient has a MDR-TB (i.e., resistance to both INH and RIF). Preventive treatment with fluoroquinolones alone or with a second drug has proved to be a good option in such cases. This option has been endorsed by WHO, CDC, and other medical societies [239,257,258].

Another shorter regimen in trials for TBI is 1-month daily weekly INH + RPT (30 doses) (1HP). Overall, 1HP was not inferior compared to 9H in preventing TB in high-risk populations with HIV, with lower adverse events and being more likely to complete treatment [247]. Although treatment with 1HP will cause an additional cost of $4655 per 1000 patients than 3HP under the assumption of equal efficacy, 1HP had the potential to be cost-effective taking into consideration the difference in completion rates, the difference in efficacy, TBI prevalence, and the price of rifapentine [259].

Although there are guidelines for standard therapy for TBI, their practical application in low- and middle-income countries with high TB incidence is challenging due to resource limitations; barriers to accessing TB care such as patient travel distances to centralized services, and indirect and direct costs associated with TB treatment for patients; in addition to constraints in the operation such as limited staff capacity, and the need for capacity building in operational research and health systems management [260].

### 6.2. TBI Treatment in MDR Strains

The current standard drug regimens with INH, RIF, and/or RPT are only suitable for drug-sensitive TB. Although there are no specific guidelines on the management of latent MDR-TB, a 6-12 regimen with fluoroquinolones, with or without a second drug depending on the susceptibility of the index patient, is suggested by WHO, CDC and some relevant medical societies such as IDSA (Infectious Diseases Society of America), ATS (American Thoracic Society), and ERS (European Respiratory Society), among others [239,258]. However, previous regimens that included pyrazinamide with fluoroquinolones or ethambutol have been abandoned due to their high incidence of adverse reactions [261,262]. 

The Chuuk TB program in Micronesia offered fluoroquinolone-based TBI regimens as a preventive treatment for contacts of MDR-TB patients. Based on MDR-TB index patients’ drug susceptibility testing results, the following drugs were prescribed to the latent contacts for 12 months. Contacts to patients with Mtb resistant to INH, RIF, and ethionamide (ETH) were treated with daily oral moxifloxacin (MFX) and ethambutol (EMB) in adults >12 years; and daily oral LVX and EMB in children ≤12 years. Contacts to patients with Mtb resistant to INH, RIF, PZA, EMB, and streptomycin (SM) isolates were treated with daily oral MFX in adults >12 years; and daily oral LVX and ETH in children ≤12 years. The observation of 104 contacts of MDR-TB who received fluoroquinolone-based TBI regimens showed that none of them developed MDR-TB after a 36-month follow-up, while 3 out of 15 who refused TBI treatment developed MDR-TB. Four of the contacts discontinued MFX treatment due to adverse effects such as nausea, gastrointestinal disturbances, muscle and joint pain, or hepatitis. Upon substituting the medication with LVX, the treatment plan continues, and it was completed [263].

Several randomized controlled trials (RCTs) are ongoing to prove the efficacy in preventing ATB of different TBI regimens in patients with presumed MDR-TB infection. Currently, in Vietnam, a RCT, known as the V-QUIN MDR trial, is in progress to evaluate the effectiveness of LVX vs. placebo treatment in latent MDR-TB in preventing the development of MDR-TB [264]. Similar to the VQUIN MDR trial, another ongoing RCT, the TB-CHAMP trial, is taking place in South Africa, analyzing LVX for the treatment of children aged <5 years in contact with adults with MDR-TB [265]. Finally, the multicenter ACTG A5300/IMPAACT I2003 PHOENIx trial investigates the efficacy of 26 weeks of delamanid (DLM, a mycolic acid synthesis inhibitor) vs. 26 weeks of INH [266].

## 7. Conclusions and Perspectives

**Figure 2:**
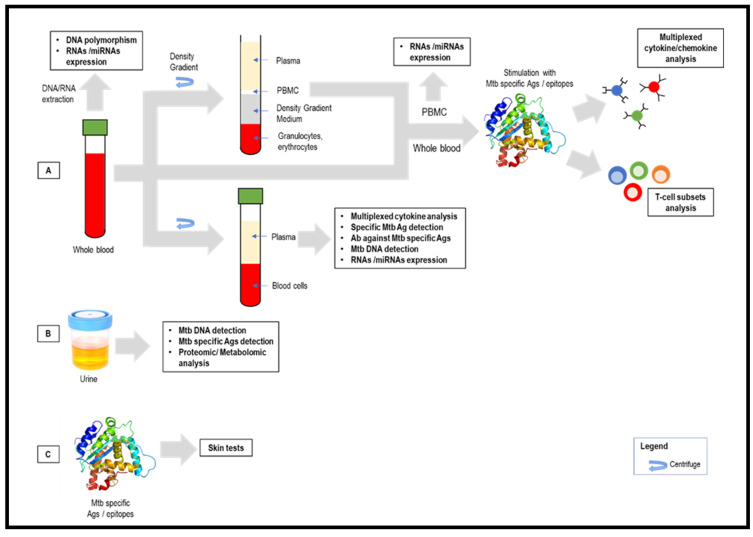
Summary of proposed tests for TBI diagnosis improvement using blood (**A**), urine (**B**), and Mtb specific skin tests (**C**).

Screening for TBI remains challenging due to the limitations of current diagnostic tests, leading to overdiagnosis and misdiagnosis. Some of the key aspects to be considered for future development of diagnostic tests for differentiating between LTBI, incipient, subclinical, and ATB suitable for children, elderly, and high-risk populations are (Figure 2):

- Unstimulated and stimulated multiplexed cytokine analysis instead of standalone marker-based on IFN-γ.
- Addition of Mtb latency Ags as stimulating Ags, and optimization of Ag stimulating time (from 24 to 72 h).
- Screening on TBI serum for Mtb Ags and specific Abs to Mtb secreted and latency Ags for the development of rapid diagnostic kits.
- Use of flow cytometry for simultaneous detection of T cell subsets and their signature cytokines.
- Study on mRNAs and microRNAs as diagnostics and therapeutics candidates for TB.
- Identification of markers not only for diagnostic purposes, but also able to assess the TB progression or reactivation risk.
- A non-invasive approach using urine for the detection of Mtb or host-related biomarkers.
- Use of Mtb-specific Ags or epitopes for development of skin test reagents.

Once the diagnosis is made, it is important to determine the drug susceptibility between the ATB index case and contacts to implement standard or individualized treatment. There is a need to revise current therapeutic schemes, particularly for TBI produced by MDR- and XDR-TB. These strains are less or not susceptible to the standard drug TBI treatment. Based on the growing trend of drug-resistant tuberculosis (DR-TB), this will become a threat to TBI control. 

Before prescribing TBI medications, it is important to assess the risk of reactivation and hepatotoxicity. Regardless of therapy, it is mandatory to minimize risks during treatment and increase patient compliance with direct observation short-course treatment. 

In conclusion, development of sensitive and specific diagnostic tests that could accurately identify the risk of progression to ATB, implementation of safer and shorter treatments, and use effective treatments against DR-TB are necessary for TBI control to contribute to achieve a world free of TB.

## Figures and Tables

**Table 1:** Diagnostic methods for TBI and ATB endorsed by WHO.

Tuberculosis Infection (TBI)	Active Tuberculosis (ATB)
**Skin tests [19]:** ○ Tuberculin Skin Test (TST) (RT23 PPD or PPD-S) ○ Cy-Tb (Serum Institute of India, India) ○ Diaskintest^®^ (Generium, Russian Federation) ○ C-TST (formerly known as ESAT6-CFP10 test, Anhui Zhifei Longcom, China) **Interferon-gamma release assays [20]:** ○ QuantiFERON^®^-TB Gold-In-Tube (Qiagen, Hilden, Germany) ○ QuantiFERON^®^-TB Gold Plus (Qiagen, Hilden, Germany) ○ T-SPOT^®^.TB (Oxford Immunotec Ltd., Milton, UK) ○ Beijing Wantai’s TB-IGRA (WanTai Biological Pharmacy Enterprise Co., Ltd., Beijing, China)	**Microscopy [21]:** ○ Ziehl–Neelsen microscopy ○ Light-emitting diode (LED) fluorescence microscopy **Culture [21]:** ○ Solid media (Löwenstein–Jensen) ○ Liquid media **Nucleic acid amplification tests [22]:** ○ Xpert^®^ MTB/RIF, Xpert^®^ MTB/RIF Ultra, and Xpert^®^ MTB/XDR (Cepheid) ○ Truenat™ (Molbio) ○ Abbott RealTime MTB and Abbott RealTime MTB RIF/INH (Abbott) ○ BD MAX™ MDR-TB (Becton Dickinson) ○ cobas^®^ MTB and cobas MTB-RIF/INH (Roche) ○ FluoroType^®^ MTBDR and FluoroType^®^ MTB (Hain Lifescience/Bruker) ○ TB-LAMP (Eiken) **Line probe assays [22]:** ○ GenoType^®^ MTBDRplus v1 and v2; GenoType^®^ MTBDRsl, (Hain Lifescience/Bruker) ○ Genoscholar™ NTM+MDRTB II; Genoscholar™ PZA-TB II (Nipro) **Lateral flow [22]:** ○ Alere Determine™ TB LAM Ag (Alere)

**Table 2:** Differences between TST, QFT^®^, and T-SPOT^®^.TB.

Criteria	TST	QFT^®^	T-SPOT^®^.TB
**Sample**	Skin test	Whole blood (Processed within 16 h)	PBMCs (Processed within 8 to 32 h)
**Responses**	Delayed-type hypersensitivity	T cells immune responses	
**Frequency of patient visit**	Two times visit	One time visit	
**Sample processing step before the test**	None	None	Isolation of PBMCs from whole blood and cell counting
**Antigens**	PPD	QFT®-GIT: ESAT-6, CFP-10, and TB7.7 QFT®-Plus: TB1-ESAT-6 and CFP-10 TB2-ESAT-6, CFP-10, and 6 short peptides	PA: ESAT-6 PB: CFP-10
**Protocol**	0.1 mL (5 IU) tuberculin injection at forearm	1 mL of blood for individual tubes	250,000 ± 50,000 cells/well
**Platform**	Induration	Enzyme-linked immunosorbent assay (ELISA)	Enzyme-linked immunospot assay (ELISPOT)
**Principle**	Measure size of induration after intradermal PPD inoculation	Quantify amount of INF-γ released by CD4 and CD8 T cells	Count number of cells that release IFN-γ
**Maximum number of samples per run**	Individual	QFT®-GIT: 28 samples; QFT®-Plus: 22 samples in a 96-well microtiter plate	24 samples in a 96-well microtiter plate
**Equipment**	Ruler	Incubator 37 °C ELISA reader	Incubator 37 °C with 5% CO_2_ Magnifying glass/Microscope
**Results interpretation**	* Positive: ≥5 mm, ≥10 mm, or ≥15 mm of induration * Negative: <5 mm, <10 mm, or <15 mm of induration * Depending on risk	Positive: ≥0.35 IU/mL (PC-NC = Any) Negative: <0.35 IU/mL (PC-NC ≥ 0.5 IU/mL) Indeterminate: <0.35 or ≥0.35 IU/mL (PC-NC < 0.5 IU/mL)	Positive: ≥8 spots Borderline: 5–8 spots Negative: ≤4 spots
**Indeterminate/Invalid**	-	NC > 8 IU/mL PC-NC < 0.5 IU/mL	NC > 10 spots PC < 20 spots
**False-positive**	BCG vaccination and NTM infections	Results not affected by BCG vaccination and most NTM infections with exception of *M. kansasii*, *M. szulgai*, and *M. marinum*	
**Turnaround time**	48 to 72 h	24 h	
**Interpretation**	Subjective	Not affected by bias	Subjective

PPD: Purified Protein Derivative; QFT^®^-GIT: Quantiferon^®^-TB Gold-In-Tube; QFT^®^-Plus: Quantiferon^®^-TB Plus; PA: Panel A (ESAT-6); PB: Panel B (CFP-10); PC: Positive control; NC: Negative Control; ESAT-6: Early Secreted Antigenic Target 6 kDa protein; CFP-10: Culture Filtrate Protein 10 KDa protein; * Skin test interpretation depends on measurement in millimeters of the induration and a person’s risk of TBI or risk of progression to ATB if infected.

**Table 3:** Cytokines associated with TB infection.

Antigen	Participants	Sample	Stimulating Time	Assay	Cytokines/Chemokines Associated to TB Infection	Cytokines/Chemokines to Distinguish TBI and Active TB	Ref.
**PPD (10 μg/mL)**	5–85 years old	PBMCs	24 h	Bio-Plex Pro Human Cytokine 27-plex Assay (Bio-Rad, Hercules, CA, USA)	Stimulated: TB > HC: IL-2 > IL-10 > IP-10	Stimulated: TBI > ATB: AUC IL-2 (0.7633) > IP-10 (0.7622) > IFN-γ (0.7128) > TNF-α (0.6878) > IL-10 (0.6822)	[117]
**ESAT-6+CFP-10 (10 μg/mL)**	15–74 years old	Whole blood	20–24 h	Bio-Plex Pro Human Cytokine 27-plex Assay (Bio-Rad, Hercules, CA, USA)	Unstimulated: TB > non-TB: VEGF > IP-10 > MCP-1 > IL-12 > MIP-1b > IFN-γ Stimulated: TB > non-TB: IP-10 > IL-2 > IL-1ra > MCP-1 > IFN-γ > IL-15	Unstimulated: ATB > TBI: VEGF (0.8106) > IP-10 (0.7717) > IL-12 (0.7476) > IFN-γ (0.7276) Stimulated: ATB > TBI: AUC PDGF-BB (0.7686)	[118]
**ESAT-6 (10 μg/mL), CFP-10 (10 μg/mL), or PPD (20 μg/mL)**	24–58 years old	Whole blood	19 h	8-plex human cytokine assay (Bio-Rad, Hercules, CA, USA)	Stimulated: ESAT-6: TB > HC: IL-2 > IP-10 > IFN-γ CFP-10: TB > HC: IL-2 > IP-10, IFN-γ PPD: TB > HC: IFN-γ > IL-2 > IP-10	Stimulated: No statistically significant, but: ESAT-6: ATB > TBI: TNF-α CFP-10: ATB > TBI: TNF-α and IL-1ra PPD: ATB > TBI: TNF-α, IL-1ra and IL-10	[119]
**ESAT-6 (10 μg/mL), CFP-10 (10 μg/mL), or PPD (20 μg/mL)**	<18 years old	Whole blood	20–24 h	17-plex, Milliplex human cytokine/chemokine kits (Millipore Corp., Billerica, MA, USA)	Stimulated: TB > non-TB: Sensitivity and specificity of IP-10, TNF-α, and IL-2 close to/exceed IFN-γ	Stimulated: ESAT-6: ATB > TBI: TNF-α and IL-1ra CFP-10: ATB > TBI: TNF-α PPD: ATB > TBI: IL-1ra and IL-10 PPD: Combination: TNF-a/IL-1ra (95.5%), and TNF-a/IL-10 (100%)	[120]
**ESAT6, CFP10, and MTB7.7-coated polyester beads**	PTB and HCW (TST^+^ and TST^−^), Adults	Whole blood	Overnight	Human XL Cytokine Discovery Premixed 10-plex kit (R&D Systems, Minneapolis, MN, USA)	Stimulated: PTB > TST^−^: IL2 and CCL11 (>80% S&S); IFN-γ+IL2 and IP10+IL2 (>90% S&S); IFN-γ+IP10+IL2, IP10+IL2+CCL11, and IFN-γ+CCL11+IL2 (>90% S&S)	Stimulated: PTB > TST^+^: IL2 and CCL11 (>80% S&S); IFN-γ+IL2 and IP10+IL2 (>90% S&S); IFN-γ+IP10+IL2 (>90% S&S, AUC = 0.94)	[114]
**QFT^®^-GIT**	21–55 years old	Whole blood	16–24 h	Bio-Plex Pro Human Cytokine 27-plex Assay (Bio-Rad, Hercules, CA, USA)	-	Unstimulated: ATB > TBI: IL-1ra (0.88) > MCP-1 (0.87) > IL-15 (0.86) > IL-12 (0.85), and IL-10 (0.84) Stimulated: ATB < TBI: IL-10 (0.8120) ATB > TBI: IFN-γ (0.7842) > MCP-1 (0.7419) > IL-1ra (0.7375)	[121]
**QFT^®^-GIT**	15–89 years old	Whole blood	16–24 h	29-plex, Milliplex human cytokine/chemokine kits (Millipore Corp., Billerica, MA, USA)	Stimulated: TB > HC: IL-2 > IFN-γ > IP-10 > IL-1RA > IL-3 > MIP-1β > GM-CSF > IL-13	Unstimulated: ATB > TBI: VEGF (0.7861) > TNF-α (0.7093) Stimulated: TBI > TB: VEGF (0.7870) and IL-5 (0.7472)	[122]
**QFT^®^-GIT**	10–60 years old	Whole blood	Overnight	29-plex LINCO-plex^®^ kits (Millipore, St. Charles, MO, USA)	-	Unstimulated: ATB > TBI: EGF (0.88) > sCD40L (0.84) Stimulated: TBI > ATB: EGF (0.90) > MIP-1β (0.79)	[123]
**QFT^®^-GIT**	17–84 years old	Whole blood	16–24 h	48-plex, Bio-Plex platform (Bio-Rad)	Unstimulated: TB > non-TB: IL-1β, IL-12-p70, IL-2, IL-8, MCP-1, PDGF-BB, VEGF, LIF Stimulated: TBI > non-TB: IL2, IP10, IFN-γ, IL13, MIG, SCF, b-NGF, IL12-p40, TRAIL, IL2 Ra, LIF ATB > non-TB: IL2, IP10, IFN-γ, MIG, SCF, b-NGF, IL12-p40, TRAIL, IL2Ra, MIF, TNF-β, IL3, IFN-α2, LIF	Seven biomarkers: MCP-1_Nil_, LIF_Nil_, IL2, IFN-γ, MIF, TRAIL, 1L2Ra-ATB (88.89%), TBI (82.35%), Non-TB (90%) Six biomarkers: MCP-1_Nil_, LIF_Nil_, IL2, IFN-γ, MIF, TRAIL-ATB (88.89%), TBI (70.59%), Non-TB (90%)	[124]
**QFT^®^-GIT**	Healthcare workers (HCW) and community control (CC), >18 years old	Whole blood	Overnight	Procartaplex11-plex (Life Technologies, Thermo Fisher Scientific, Waltham, MA, USA)	-	Stimulated: HCW^LTB+^ and CC^LTB+^ > HCW^LTB−^ and CC^LTB−^: IFN-γ and IL-2 HCW^LTB+^ > HCW^LTB−^ and CC^LTB−^: IL-8 HCW^LTB+^ and CC^LTB+^ > CC^LTB−^: IL-5 HCW^LTB+^ > HCW^LTB−^: IL-5 and IL-10	[125]

**Table 4:** mRNAs and miRNAs that significantly differentiate ATB and TBI.

Participants	Sample	Assay	Results	Ref.
**Adults**	Whole blood	Microarray	Differentiate ATB from TBI: CCR2 (1.00), MSR1 (0.99), C1QB (0.97), MAPK14 (0.95), LILRB4 (0.94), C2 (0.92), and CCRL2 (0.86)	[172]
**Adults**	Whole blood	RNA-Seq	Differentiate ATB from TBI: ANKRD22, APOL4, BANK1, BATF2, DHRS9, DOCK9, EPHA4, ETV7, FAM26, FMN1, NPC2, NT5E, and WARS High AUC: DOCK9 (0.982), NPC2 (0.976), and EPHA4 (0.940)	[173]
**Adults**	Serum	RNA-Seq	Combination analysis of hsa-let-7i-5p, miR-122-5p, miR-148a-3p, miR-151a-3p, miR-21-5p, miR-423-5p, miR-451a, and miR-486-5p able to classify ATB and TBI with accuracy of 71.8%	[174]
**Adults**	PBMCs	Microarray	Differentiate ATB from TBI: hsa-miR-130b, hsa-miR-21, hsa-miR-223, hsa-miR-302a, hsa-miR-424, hsa-miR-451, and hsa-miR-486-5p	[175]
**Adults**	PBMCs	Microarray	Differentiate ATB from TBI: hsa-miR-146a-5pb-ST20 hsa-miR-150-5pb-CPD, ARRB2, FFAR2, NUP214, PNMA3, C20orf24, C16orf57 hsa-miR-16-5p-CPD, C15orf39, C16orf57, TUBA1A hsa-miR-221-3p-ANXA1, FOS, PLAUR, TIMP2, C16orf57, MDN	[176]

## Data Availability

Not applicable.

## References

[1] WHO (2021). Global Tuberculosis Report 2021: Incidence of Tuberculosis (per 100,000 people). The World Bank. https://data.worldbank.org/indicator/SH.TBS.INCD?most_recent_value_desc=true.

[2] Floyd K., Glaziou P., Zumla A., Raviglione M. (2018). The global tuberculosis epidemic and progress in care, prevention, and research: An overview in year 3 of the End TB era. Lancet Respir. Med..

[3] Rao M., Ippolito G., Mfinanga S., Ntoumi F., Yeboah-Manu D., Vilaplana C., Zumla A., Maeurer M. (2019). Improving treatment outcomes for MDR-TB–Novel host-directed therapies and personalised medicine of the future. Int. J. Infect. Dis..

[4] WHO (2020). Fact Sheets: Tuberculosis.

[5] Silva S., Arinaminpathy N., Atun R., Goosby E., Reid M. (2021). Economic impact of tuberculosis mortality in 120 countries and the cost of not achieving the Sustainable Development Goals tuberculosis targets: A full-income analysis. Lancet Glob. Health.

[6] Alliance T. Drug resistance: A response to Antimicrobial Resistance Includes Tackling TB. https://www.tballiance.org/why-new-tb-drugs/antimicrobial-resistance.

[7] WHO (2022). WHO Consolidated Guidelines on Tuberculosis: Module 3: Diagnosis: Tests for TB Infection.

[8] Cohen A., Mathiasen V.D., Schön T., Wejse C. (2019). The global prevalence of latent tuberculosis: A systematic review and meta-analysis. Eur. Respir. J..

[9] Boom W.H., Schaible U.E., Achkar J.M. (2021). The knowns and unknowns of latent Mycobacterium tuberculosis infection. J. Clin. Investig..

[10] Ai J.-W., Ruan Q.-L., Liu Q.-H., Zhang W.-H. (2016). Updates on the risk factors for latent tuberculosis reactivation and their managements. Emerg. Microbes Infect..

[11] Houben R.M.G.J., Dodd P.J. (2016). The global burden of latent tuberculosis infection: A re-estimation using mathematical modelling. PLoS Med..

[12] Knight G.M., McQuaid C.F., Dodd P.J., Houben R.M.G.J. (2019). Global burden of latent multidrug-resistant tuberculosis: Trends and estimates based on mathematical modelling. Lancet Infect. Dis..

[13] Reichler M.R., Khan A., Yuan Y., Chen B., McAuley J., Mangura B., Sterling T.R., Team T.E.S.C.T.O. (2020). Duration of exposure among close contacts of patients with infectious tuberculosis and risk of latent tuberculosis infection. Clin. Infect. Dis..

[14] WHO (2018). Latent Tuberculosis Infection: Updated and Consolidated Guidelines for Programmatic Management.

[15] Hamada Y., Cirillo D.M., Matteelli A., Penn-Nicholson A., Rangaka M.X., Ruhwald M. (2021). Tests for tuberculosis infection: Landscape analysis. Eur. Respir. J..

[16] Force U.P.S.T. (2016). Screening for latent tuberculosis infection in adults: US Preventive Services Task Force Recommendation Statement. JAMA.

[17] Stein C.M., Nsereko M., Malone L.L., Okware B., Kisingo H., Nalukwago S., Chervenak K., Mayanja-Kizza H., Hawn T.R., Boom W.H. (2019). Long-term stability of resistance to latent Mycobacterium tuberculosis infection in highly exposed tuberculosis household contacts in Kampala, Uganda. Clin. Infect. Dis..

[18] Verrall A.J., Alisjahbana B., Apriani L., Novianty N., Nurani A.C., van Laarhoven A., Ussher J.E., Indrati A., Ruslami R., Netea M.G. (2020). Early clearance of Mycobacterium tuberculosis: The INFECT case contact cohort study in Indonesia. J. Infect. Dis..

[19] WHO (2022). Rapid Communication: TB Antigen-Based Skin Tests for the Diagnosis of TB Infection.

[20] WHO (2022). Use of Alternative Interferon-Gamma Release Assays for the Diagnosis of TB Infection: WHO Policy Statement.

[21] WHO (2015). Implementing Tuberculosis Diagnostics: Policy Framework.

[22] WHO (2021). WHO Consolidated Guidelines on Tuberculosis: Module 3: Diagnosis: Rapid Diagnostics for Tuberculosis Detection, 2021 Update.

[23] CDC (2020). Fact Sheets: Tuberculin Skin Testing.

[24] Nayak S., Acharjya B. (2012). Mantoux test and its interpretation. Indian Dermatol. Online J..

[25] van Pinxteren Laurens A.H., Ravn P., Agger Else M., Pollock J., Andersen P. (2000). Diagnosis of tuberculosis based on the two specific antigens ESAT-6 and CFP10. Clin. Diagn. Lab. Immunol..

[26] Ruhwald M., Aggerbeck H., Gallardo R.V., Hoff S.T., Villate J.I., Borregaard B., Martinez J.A., Kromann I., Penas A., Anibarro L.L. (2017). Safety and efficacy of the C-Tb skin test to diagnose Mycobacterium tuberculosis infection, compared with an interferon γ release assay and the tuberculin skin test: A phase 3, double-blind, randomised, controlled trial. Lancet Respir. Med..

[27] Weldingh K., Andersen P. (2008). ESAT-6/CFP10 skin test predicts disease in M. tuberculosis-infected guinea pigs. PLoS ONE.

[28] Arend S.M., Franken W.P.J., Aggerbeck H., Prins C., van Dissel J.T., Thierry-Carstensen B., Tingskov P.N., Weldingh K., Andersen P. (2008). Double-blind randomized Phase I study comparing rdESAT-6 to tuberculin as skin test reagent in the diagnosis of tuberculosis infection. Tuberculosis.

[29] Bergstedt W., Tingskov P.N., Thierry-Carstensen B., Hoff S.T., Aggerbeck H., Thomsen V.O., Andersen P., Andersen A.B. (2010). First-in-man open clinical trial of a combined rdESAT-6 and rCFP-10 tuberculosis specific skin test reagent. PLoS ONE.

[30] Slogotskaya L., Litvinov V., Kochetkov Y., Ovsyankina E., Kudlay D., Seltsovsky P., Nikolenko N., Ivanova D. (2012). New skin test with recombinant protein CFP10-ESAT6 in patients (children and adults) with tuberculosis, non-tuberculosis disease and latent TB infection. Eur. Respir. J..

[31] Slogotskaya L., Bogorodskaya E., Ivanova D., Makarova M., Guntupova L., Litvinov V., Seltsovsky P., Kudlay D., Nikolenko N. (2013). Sensitivity and specificity of new skin test with recombinant protein CFP10-ESAT6 in patients with tuberculosis and individuals with non- tuberculosis diseases. Eur. Respir. J..

[32] Slogotskaya L., Bogorodskaya E., Ivanova D., Sevostyanova T. (2018). Comparative sensitivity of the test with tuberculosis recombinant allergen, containing ESAT6-CFP10 protein, and Mantoux test with 2 TU PPD-L in newly diagnosed tuberculosis children and adolescents in Moscow. PLoS ONE.

[33] Hoff S.T., Peter J.G., Theron G., Pascoe M., Tingskov P.N., Aggerbeck H., Kolbus D., Ruhwald M., Andersen P., Dheda K. (2016). Sensitivity of C-Tb: A novel RD-1-specific skin test for the diagnosis of tuberculosis infection. Eur. Respir. J..

[34] Hanif S., R A.-A., AS M. (2010). Species-specific antigenic Mycobacterium tuberculosis proteins tested by delayed-type hypersensitivity response. Int. J. Tuberc. Lung Dis..

[35] Kalra M., Khuller G.K., Sheikh J.A., Verma I. (2010). Evaluation of Mycobacterium tuberculosis specific RD antigens for delayed type hypersensitivity responses in guinea pig. Indian J. Exp. Biol..

[36] Lyashchenko K., Manca C., Colangeli R., Heijbel A., Williams A., Gennaro Maria L. (1998). Use of Mycobacterium tuberculosis complex-specific antigen cocktails for a skin test specific for tuberculosis. Infect. Immun..

[37] Luo W., Qu Z.-L., Xie Y., Xiang J., Zhang X.-L. (2015). Identification of a novel immunodominant antigen Rv2645 from RD13 with potential as a cell-mediated immunity-based TB diagnostic agent. J. Infect..

[38] Aagaard C., Brock I., Olsen A., Ottenhoff T.H.M., Weldingh K., Andersen P. (2004). Mapping immune reactivity toward Rv2653 and Rv2654: Two novel low-molecular-mass antigens found specifically in the Mycobacterium tuberculosis complex. J. Infect. Dis..

[39] Mori T., Sakatani M., Yamagishi F., Takashima T., Kawabe Y., Nagao K., Shigeto E., Harada N., Mitarai S., Okada M. (2004). Specific detection of tuberculosis infection. Am. J. Respir. Crit. Care Med..

[40] CDC (2021). Interferon Gamma Release Assay Testing for Latent Tuberculosis Infection: Physician Guidelines.

[41] CDC (2016). Interferon-Gamma Release Assays (IGRAs)—Blood Tests for TB Infection.

[42] Ronge L., Sloot R., Du Preez K., Kay A.W., Kirchner H.L., Grewal H.M.S., Mandalakas A.M., Hesseling A.C. (2021). The magnitude of interferon gamma release assay responses in children with household tuberculosis contact is associated with tuberculosis exposure and disease status. Pediatr. Infect. Dis. J..

[43] Buonsenso D., Delogu G., Perricone C., Grossi R., Careddu A., De Maio F., Palucci I., Sanguinetti M., Valentini P., Sali M. (2020). Accuracy of QuantiFERON-TB Gold Plus test for diagnosis of Mycobacterium tuberculosis infection in children. J. Clin. Microbiol..

[44] Surve S., Bhor V., Naukariya K., Begum S., Munne K., Tipre P., Sutar N., Jaiswal A., Bhonde G., Chauhan S. (2021). Discordance between TST and QFT-TBGold Plus for latent tuberculosis screening among under-five children: An interim analysis. J. Trop. Pediatr..

[45] Carranza C., Pedraza-Sanchez S., de Oyarzabal-Mendez E., Torres M. (2020). Diagnosis for latent tuberculosis infection: New alternatives. Front. Immunol..

[46] Nguyen D.T., Teeter L.D., Graves J., Graviss E.A. (2018). Characteristics associated with negative interferon-γ release assay results in culture-confirmed tuberculosis patients, Texas, USA, 2013–2015. Emerg. Inf. Dis..

[47] Qiagen (2016). QuantiFERON-TB Gold Plus (QFT-Plus) ELISA Package Insert. http://www.quantiferon.com/wp-content/uploads/2017/04/English_QFTPlus_ELISA_R04_022016.pdf.

[48] Pérez-Recio S., Pallarès N., Grijota-Camino Maria D., Sánchez-Montalvá A., Barcia L., Campos-Gutiérrez S., Pomar V., Rabuñal-Rey R., Balcells María E., Gazel D. (2021). Identification of recent tuberculosis exposure using QuantiFERON-TB Gold Plus, a multicenter study. Microbiol. Spectr..

[49] Kellar K.L., Gehrke J., Weis S.E., Mahmutovic-Mayhew A., Davila B., Zajdowicz M.J., Scarborough R., LoBue P.A., Lardizabal A.A., Daley C.L. (2011). Multiple cytokines are released when blood from patients with tuberculosis is stimulated with Mycobacterium tuberculosis antigens. PLoS ONE.

[50] Hoffmann H., Avsar K., Göres R., Mavi S.C., Hofmann-Thiel S. (2016). Equal sensitivity of the new generation QuantiFERON-TB Gold plus in direct comparison with the previous test version QuantiFERON-TB Gold IT. Clin. Microbiol. Infect..

[51] Winglee K., Hill A.N., Belknap R., Stout J.E., Ayers T.L. (2022). Variability of interferon-γ release assays in people at high risk of tuberculosis infection or progression to tuberculosis disease living in the United States. Clin. Microbiol. Infect..

[52] Moon H.W., Gaur R.L., Tien S.S., Spangler M., Pai M., Banaei N. (2017). Evaluation of QuantiFERON-TB Gold-Plus in health care workers in a low-incidence setting. J. Clin. Microbiol..

[53] Nemes E., Rozot V., Geldenhuys H., Bilek N., Mabwe S., Abrahams D., Makhethe L., Erasmus M., Keyser A., Toefy A. (2017). Optimization and interpretation of serial quantiferon testing to measure acquisition of Mycobacterium tuberculosis infection. Am. J. Respir. Crit. Care Med..

[54] Nienhaus A., Ringshausen F.C., Costa J.T., Schablon A., Tripodi D. (2013). IFN-γ release assay versus tuberculin skin test for monitoring TB infection in healthcare workers. Expert Rev. Anti. Infect. Ther..

[55] Brown J., Kumar K., Reading J., Harvey J., Murthy S., Capocci S., Hopkins S., Seneviratne S., Cropley I., Lipman M. (2017). Frequency and significance of indeterminate and borderline Quantiferon Gold TB IGRA results. Eur. Respir. J..

[56] Jonsson J., Westman A., Bruchfeld J., Sturegård E., Gaines H., Schön T. (2017). A borderline range for Quantiferon Gold In-Tube results. PLoS ONE.

[57] Anibarro L., Trigo M., Villaverde C., Pena A., Cortizo S., Sande D., Pazos R., Gonzalez-Fernandez A. (2011). Interferon-gamma release assays in tuberculosis contacts: Is there a window period?. Eur. Respir. J..

[58] Fox G.J., Barry S.E., Britton W.J., Marks G.B. (2013). Contact investigation for tuberculosis: A systematic review and meta-analysis. Eur. Respir. J..

[59] Stieber F., Howard J., Manissero D., Boyle J., Ndunda N., Love J., Yang M., Schumacher A., Uchiyama R., Parsons S. (2021). Evaluation of a lateral-flow nanoparticle fluorescence assay for TB infection diagnosis. Int. J. Tuberc. Lung Dis..

[60] Fukushima K., Akagi K., Kondo A., Kubo T., Sakamoto N., Mukae H. (2022). First clinical evaluation of the QIAreachTM QuantiFERON-TB for tuberculosis infection and active pulmonary disease. Pulmonology.

[61] Saluzzo F., Mantegani P., Poletti de Chaurand V., Cirillo D.M. (2022). QIAreach QuantiFERON-TB for the diagnosis of Mycobacterium tuberculosis infection. Eur. Respir. J..

[62] Kaaba C., Ruperez M., Kosloff B., Ndunda N., Shanaube K., Ayles H. (2021). Assessing usability of QIAreach QuantiFERON-TB platform in a high tuberculosis prevalence, low-resource setting. ERJ Open Res..

[63] Immunotec O. (2019). T-SPOT®.TB: Package Insert. https://www.tspot.com/wp-content/uploads/2020/01/The-T-SPOT.TB-test-package-insert.pdf.

[64] Janssens J.-P., Roux-Lombard P., Perneger T., Metzger M., Vivien R., Rochat T. (2007). Quantitative scoring of an interferon-γ assay for differentiating active from latent tuberculosis. Eur. Respir. J..

[65] Rego K., Pereira K., MacDougall J., Cruikshank W. (2018). Utility of the T-SPOT. Tuberculosis.

[66] Immunotec O. (2022). T-Cell Select™. https://www.oxfordimmunotec.com/international/products-services/t-cell-select/.

[67] Wantai WANTAI TB-IGRA: Diagnostic Kit for T Cell Infected with Mycobacterium Tuberculosis. http://www.ystwt.cn/wp-content/uploads/2018/04/Wantai-TB-IGRA.pdf.

[68] Li G., Li F., Zhao H.-M., Wen H.-L., Li H.-C., Li C.-L., Ji P., Xu P., Wu K., Hu Z.-D. (2017). Evaluation of a new IFN-γ release assay for rapid diagnosis of active tuberculosis in a high-incidence setting. Front. Cell. Infect. Microbiol..

[69] Della Bella C., Spinicci M., Alnwaisri H.F.M., Bartalesi F., Tapinassi S., Mencarini J., Benagiano M., Grassi A., D’Elios S., Troilo A. (2020). LIOFeron^®^TB/LTBI: A novel and reliable test for LTBI and tuberculosis. Int. J. Infect. Dis..

[70] Lewinsohn D.M., Leonard M.K., LoBue P.A., Cohn D.L., Daley C.L., Desmond E., Keane J., Lewinsohn D.A., Loeffler A.M., Mazurek G.H. (2017). Official American Thoracic Society/Infectious Diseases Society of America/Centers for Disease Control and Prevention Clinical Practice Guidelines: Diagnosis of Tuberculosis in Adults and Children. Clin. Infect. Dis..

[71] McKenna L., Sari A.H., Mane S., Scardigli A., Brigden G., Rouzier V., Becerra M.C., Hesseling A.C., Amanullah F. (2022). Pediatric tuberculosis research and development: Progress, priorities and funding opportunities. Pathogens.

[72] Mazurek G.H., Jereb J., Vernon A., LoBue P., Goldberg S., Castro K. (2010). Updated guidelines for using Interferon Gamma Release Assays to detect Mycobacterium tuberculosis infection—United States, 2010. MMWR Recomm. Rep..

[73] Soler-Garcia A., Gamell A., Pérez-Porcuna T., Soriano-Arandes A., Santiago B., Tórtola T., Ruiz-Serrano M.J., Korta Murua J.J., Bustillo-Alonso M., Garrote-Llanos M.I. (2021). Performance of QuantiFERON-TB Gold Plus assays in children and adolescents at risk of tuberculosis: A cross-sectional multicentre study. Thorax.

[74] Yun K.W., Kim Y.K., Kim H.R., Lee M.K., Lim I.S. (2016). Usefulness of interferon-γ release assay for the diagnosis of latent tuberculosis infection in young children. Korean J. Pediatr..

[75] Kay A.W., Islam S.M., Wendorf K., Westenhouse J., Barry P.M. (2018). Interferon-γ release assay performance for tuberculosis in childhood. Pediatrics.

[76] Gaensbauer J., Young J., Harasaki C., Aiona K., Belknap R., Haas M.K. (2020). Interferon-gamma release assay testing in children younger than 2 years in a US-based health system. Pediatr. Infect. Dis. J..

[77] Ahmed A., Feng P.-J.I., Gaensbauer J.T., Reves R.R., Khurana R., Salcedo K., Punnoose R., Katz D.J., CONSORTIUM f.t.T.E.S. (2020). Interferon-γ release assays in children <15 years of age. Pediatrics.

[78] Wendorf K.A., Lowenthal P., Feraud J., Cabanting N., Murto C. (2020). Interferon-γ release assays for tuberculosis infection diagnosis in refugees < 5 years old. Pediatrics.

[79] Connell T.G., Ritz N., Paxton G.A., Buttery J.P., Curtis N., Ranganathan S.C. (2008). A three-way comparison of tuberculin skin testing, QuantiFERON-TB Gold and T-SPOT.TB in children. PLoS ONE.

[80] Bae W., Park K.U., Song E.Y., Kim S.J., Lee Y.J., Park J.S., Cho Y.-J., Yoon H.I., Yim J.-J., Lee C.-T. (2016). Comparison of the sensitivity of QuantiFERON-TB Gold In-Tube and T-SPOT.TB according to patient age. PLoS ONE.

[81] Scordo J.M., Aguillón-Durán G.P., Ayala D., Quirino-Cerrillo A.P., Rodríguez-Reyna E., Joya-Ayala M., Mora-Guzmán F., Ledezma-Campos E., Villafañez A., Schlesinger L.S. (2021). Interferon gamma release assays for detection of latent Mycobacterium tuberculosis in older Hispanic people. Int. J. Infect. Dis..

[82] Dheda K., Barry C.E., Maartens G. (2016). Tuberculosis. Lancet.

[83] Richeldi L., Losi M., D’Amico R., Luppi M., Ferrari A., Mussini C., Codeluppi M., Cocchi S., Prati F., Paci V. (2009). Performance of tests for latent tuberculosis in different groups of immunocompromised patients. Chest.

[84] Auguste P., Tsertsvadze A., Pink J., Court R., McCarthy N., Sutcliffe P., Clarke A. (2017). Comparing interferon-gamma release assays with tuberculin skin test for identifying latent tuberculosis infection that progresses to active tuberculosis: Systematic review and meta-analysis. BMC Infect. Dis..

[85] Fernández-Blázquez A., Argüelles Menéndez P., Sabater-Cabrera C., García-García J.M., Asensi Álvarez V., Palacios Gutiérrez J.J. (2020). Diagnosis of tuberculous infection in immunosuppressed patients and/or candidates for biologics using a combination of 2 IGRA tests: T-SPOT.TB/QuantiFERON TB Gold In-Tube vs. T-SPOT.TB/QuantiFERON TB Gold Plus. Arch. Bronconeumol..

[86] Park C.H., Park J.H., Jung Y.S. (2022). Impact of immunosuppressive therapy on the performance of latent tuberculosis screening tests in patients with inflammatory bowel disease: A systematic review and meta-analysis. J. Pers. Med..

[87] Wong S.H., Gao Q., Tsoi K.K., Wu W.K., Tam L.S., Lee N., Chan F.K., Wu J.C., Sung J.J., Ng S.C. (2016). Effect of immunosuppressive therapy on interferon γ release assay for latent tuberculosis screening in patients with autoimmune diseases: A systematic review and meta-analysis. Thorax.

[88] Fritschi N., Curtis N., Ritz N. (2020). Bacille Calmette Guérin (BCG) and new TB vaccines: Specific, cross-mycobacterial and off-target effects. Paediatr. Respir. Rev..

[89] Lange C., Aaby P., Behr M.A., Donald P.R., Kaufmann S.H.E., Netea M.G., Mandalakas A.M. (2022). 100 years of Mycobacterium bovis bacille Calmette-Guérin. Lancet Infect. Dis..

[90] Iglesias M.J., Martin C. (2015). Editorial Commentary: Nonspecific beneficial effects of BCG vaccination in high-income countries, should we extend recommendation of BCG vaccination?. Clin. Infect. Dis..

[91] de Castro M.J., Pardo-Seco J., Martinón-Torres F. (2015). Nonspecific (Heterologous) Protection of neonatal BCG vaccination against hospitalization due to respiratory infection and sepsis. Clin. Infect. Dis..

[92] Broset E., Pardo-Seco J., Kanno A.I., Aguilo N., Dacosta A.I., Rivero-Calle I., Gonzalo-Asensio J., Locht C., Leite L.C.C., Martin C. (2021). BCG vaccination improves DTaP immune responses in mice and is associated with lower pertussis incidence in ecological epidemiological studies. EBioMedicine.

[93] Domínguez J., Ruiz-Manzano J., Souza-Galvão M.D., Latorre I., Milà C., Blanco S., Jiménez M.Á., Prat C., Lacoma A., Altet N. (2008). Comparison of two commercially available gamma interferon blood tests for immunodiagnosis of tuberculosis. Clin. Vaccine Immunol..

[94] Pai M., Zwerling A., Menzies D. (2008). Systematic review: T-cell–based assays for the diagnosis of latent tuberculosis infection: An update. Ann. Intern. Med..

[95] Kurtz T., Feil A.C., Nascimento L.S., de Oliveira Abreu P., Scotta M.C., Pinto L.A. (2019). Effect of neonatal bacille Calmette-Guérin on the tuberculin skin test reaction in the first 2 years of life. Int. J. Tuberc. Lung Dis..

[96] Anibarro L., Trigo M., Villaverde C., Pena A., González-Fernández A. (2011). Tuberculin skin test and interferon-γ release assay show better correlation after the tuberculin ‘window period’ in tuberculosis contacts. Scand. J. Infect. Dis..

[97] Dowdy D.W., Behr M.A. (2022). Are we underestimating the annual risk of infection with Mycobacterium tuberculosis in high-burden settings?. Lancet Infect. Dis..

[98] Faust L., Ruhwald M., Schumacher S., Pai M. (2020). How are high burden countries implementing policies and tools for latent tuberculosis infection? A survey of current practices and barriers. Health Sci. Rep..

[99] Alyaquobi F., AlMaqbali A.A., Al-Jardani A., Ndunda N., Al Rawahi B., Alabri B., AlSadi A.M., AlBaloshi J.A., Al-Baloshi F.S., Al-Essai N.A. (2020). Screening migrants from tuberculosis high-endemic countries for latent tuberculosis in Oman: A cross sectional cohort analysis. Travel Med. Infect. Dis..

[100] Campbell J.R., Krot J., Elwood K., Cook V., Marra F. (2015). A systematic review on TST and IGRA tests used for diagnosis of LTBI in immigrants. Mol. Diagn. Ther..

[101] Elfrink F., van den Hoek A., Mensen M.E., Sonder G.J.B. (2014). Screening travellers to high-endemic countries for infection with Mycobacterium tuberculosis using interferon gamma release assay; a prospective study. BMC Infect. Dis..

[102] Spruijt I., Joren C., Schimmel H., Procee F., Alam Y., van den Hof S., Erkens C. (2022). The identification of prevalent tuberculosis disease through infection screening among high-risk migrants in the Netherlands. Eur. Respir. J..

[103] Apriani L., McAllister S., Sharples K., Alisjahbana B., Ruslami R., Hill P.C., Menzies D. (2019). Latent tuberculosis infection in healthcare workers in low- and middle-income countries: An updated systematic review. Eur. Respir. J..

[104] Swaminathan N., Perloff S.R., Zuckerman J.M. (2021). Prevention of Mycobacterium tuberculosis transmission in health care settings. Infect. Dis. Clin. N. Am..

[105] Fox G.J., Redwood L., Chang V., Ho J. (2021). The effectiveness of individual and environmental infection control measures in reducing the transmission of Mycobacterium tuberculosis: A systematic review. Clin. Infect. Dis..

[106] Mok J.H. (2016). Diagnosis and treatment of latent tuberculosis infection in healthcare workers. Tuberc. Respir. Dis..

[107] Park Y., Kim S.Y., Kim J.W., Park M.S., Kim Y.S., Chang J., Kang Y.A. (2018). Serial testing of healthcare workers for latent tuberculosis infection and long-term follow up for development of active tuberculosis. PLoS ONE.

[108] Dobler C.C., Farah W.H., Alsawas M., Mohammed K., Breeher L.E., Murad M.H., Molella R.G. (2018). Tuberculin Skin test conversions and occupational exposure risk in US healthcare workers. Clin. Infect. Dis..

[109] Sosa L.E., Njie G.J., Lobato M.N., Bamrah Morris S., Buchta W., Casey M.L., Goswami N.D., Gruden M., Hurst B.J., Khan A.R. (2019). Tuberculosis screening, testing, and treatment of U.S. health care personnel: Recommendations from the National Tuberculosis Controllers Association and CDC, 2019. MMWR Morb. Mortal. Wkly. Rep..

[110] Machingaidze S., Verver S., Mulenga H., Abrahams D.-A., Hatherill M., Hanekom W., Hussey G.D., Mahomed H. (2012). Predictive value of recent QuantiFERON Conversion for tuberculosis disease in adolescents. Am. J. Respir. Crit Care Med..

[111] Park J.H., Kim N., Park H., Kim T.S., Park S.W., Roh E.Y., Yoon J.H., Shin S. (2020). The use of a borderline zone for the interpretation of interferon-gamma release assay results for serial screening of healthcare workers. PLoS ONE.

[112] Leung W.L., Law K.L., Leung V.S.S., Yip C.W., Leung C.C., Tam C.M., Kam K.M. (2009). Comparison of intracellular cytokine flow cytometry and an enzyme immunoassay for evaluation of cellular immune response to active tuberculosis. Clin. Vaccine Immunol..

[113] El-Sheikh N., Mousa N.O., Osman A., Tawfeik A.M., Taha B.A., Mahran H., Saleh A.M., El-shiekh I., Amin W., Elrefaei M. (2021). Assessment of interferon gamma-induced protein 10 mRNA release assay for detection of latent tuberculosis infection in egyptian pediatric household contacts. Int. J. Infect. Dis..

[114] Kumar N.P., Moideen K., Banurekha V.V., Nair D., Babu S. (2019). Plasma proinflammatory cytokines are markers of disease severity and bacterial burden in pulmonary tuberculosis. Open Forum Infect. Dis..

[115] Soeroto A.Y., Dahlan Z., Kartasasmita C.B., Parwati I. (2013). Serum cytokines level can differentiate active pulmonary tuberculosis from latent TB. Eur. Respir. J..

[116] Sheffee N.S., Rubio-Reyes P., Mirabal M., Calero R., Carrillo-Calvet H., Chen S., Chin K.L., Shakimi N.A.S., Anis F.Z., Suraiya S. (2021). Engineered Mycobacterium tuberculosis antigen assembly into core-shell nanobeads for diagnosis of tuberculosis. Nanomed. Nanotechnol. Biol. Med..

[117] Robison H.M., Chapman C.A., Zhou H., Erskine C.L., Theel E., Peikert T., Lindestam Arlehamn C.S., Sette A., Bushell C., Welge M. (2021). Risk assessment of latent tuberculosis infection through a multiplexed cytokine biosensor assay and machine learning feature selection. Sci. Rep..

[118] Hur Y.-G., Kang Y.A., Jang S.-H., Hong J.Y., Kim A., Lee S.A., Kim Y., Cho S.-N. (2015). Adjunctive biomarkers for improving diagnosis of tuberculosis and monitoring therapeutic effects. J. Infect..

[119] Wu J., Wang S., Lu C., Shao L., Gao Y., Zhou Z., Huang H., Zhang Y., Zhang W. (2017). Multiple cytokine responses in discriminating between active tuberculosis and latent tuberculosis infection. Tuberculosis.

[120] Wang S., Li Y., Shen Y., Wu J., Gao Y., Zhang S., Shao L., Jin J., Zhang Y., Zhang W. (2018). Screening and identification of a six-cytokine biosignature for detecting TB infection and discriminating active from latent TB. J. Transl. Med..

[121] Clifford V., Tebruegge M., Zufferey C., Germano S., Forbes B., Cosentino L., Matchett E., McBryde E., Eisen D., Robins-Browne R. (2019). Cytokine biomarkers for the diagnosis of tuberculosis infection and disease in adults in a low prevalence setting. Tuberculosis.

[122] Tebruegge M., Dutta B., Donath S., Ritz N., Forbes B., Camacho-Badilla K., Clifford V., Zufferey C., Robins-Browne R., Hanekom W. (2015). Mycobacteria-specific cytokine responses detect tuberculosis infection and distinguish latent from active tuberculosis. Am. J. Respir. Crit. Care Med..

[123] Suzukawa M., Akashi S., Nagai H., Nagase H., Nakamura H., Matsui H., Hebisawa A., Ohta K. (2016). Combined analysis of IFN-γ, IL-2, IL-5, IL-10, IL-1RA and MCP-1 in QFT supernatant is useful for distinguishing active tuberculosis from latent infection. PLoS ONE.

[124] Won E.-J., Choi J.-H., Cho Y.-N., Jin H.-M., Kee H.J., Park Y.-W., Kwon Y.-S., Kee S.-J. (2017). Biomarkers for discrimination between latent tuberculosis infection and active tuberculosis disease. J. Infect..

[125] Chegou N.N., Black G.F., Kidd M., van Helden P.D., Walzl G. (2009). Host markers in Quantiferon supernatants differentiate active TB from latent TB infection: Preliminary report. BMC Pulm. Med..

[126] La Manna M.P., Orlando V., Li Donni P., Sireci G., Di Carlo P., Cascio A., Dieli F., Caccamo N. (2018). Identification of plasma biomarkers for discrimination between tuberculosis infection/disease and pulmonary non tuberculosis disease. PLoS ONE.

[127] Essone P.N., Leboueny M., Maloupazoa Siawaya A.C., Alame-Emane A.K., Aboumegone Biyogo O.C., Dapnet Tadatsin P.H., Mveang Nzoghe A., Essamazokou D.U., Mvoundza Ndjindji O., Padzys G.-S. (2019). *M. tuberculosis* infection and antigen specific cytokine response in healthcare workers frequently exposed to tuberculosis. Sci. Rep..

[128] Kak G., Raza M., Tiwari B.K. (2018). Interferon-gamma (IFN-γ): Exploring its implications in infectious diseases. Biomol. Concepts.

[129] Green A.M., DiFazio R., Flynn J.L. (2013). IFN-γ from CD4 T cells is essential for host survival and enhances CD8 T cell function during Mycobacterium tuberculosis infection. J. Immunol..

[130] Santos J.A., Duarte R., Nunes C. (2020). Host factors associated to false negative and indeterminate results in an interferon-γ release assay in patients with active tuberculosis. Pulmonology.

[131] Pan L., Jia H., Liu F., Sun H., Gao M., Du F., Xing A., Du B., Sun Q., Wei R. (2015). Risk factors for false-negative T-SPOT.TB assay results in patients with pulmonary and extra-pulmonary TB. J. Infect..

[132] Hang N.T.L., Lien L.T., Kobayashi N., Shimbo T., Sakurada S., Thuong P.H., Hong L.T., Tam D.B., Hijikata M., Matsushita I. (2011). Analysis of factors lowering sensitivity of interferon-γ release assay for tuberculosis. PLoS ONE.

[133] Guo J., Li Q., Zhang X., Yao C., Liu R., Pang Y., Gao M. (2021). Increased expression of IL-10 in peripheral blood mononuclear cells correlates with negative interferon-γ release assay results in culture-confirmed tuberculosis patients. Infect. Drug Resist..

[134] Yamasue M., Komiya K., Usagawa Y., Umeki K., Nureki S.I., Ando M., Hiramatsu K., Nagai H., Kadota J.I. (2020). Factors associated with false negative interferon-γ release assay results in patients with tuberculosis: A systematic review with meta-analysis. Sci. Rep..

[135] Kim K.H., Jeong N., Lim J.U., Lee H.Y., Lee J., Kim S.C., Kang J.Y. (2022). Clinical relevance of false-negative interferon-gamma release assays in patients with tuberculous pleurisy in an intermediate tuberculosis burden country. J. Thorac. Dis..

[136] Qiu B., Liu Q., Li Z., Song H., Xu D., Ji Y., Jiang Y., Tian D., Wang J. (2020). Evaluation of cytokines as a biomarker to distinguish active tuberculosis from latent tuberculosis infection: A diagnostic meta-analysis. BMJ Open.

[137] Liao W., Lin J.-X., Leonard W.J. (2013). Interleukin-2 at the crossroads of effector responses, tolerance, and immunotherapy. Immunity.

[138] Jafrasteh A., Karimi A., Hoseinialfatemi S.M., Azimi L., Tabarsi P., Nasehi M., Naseri M., Panahi Mishkar A., Sheikhi M., Mansour Ghanaie R. (2021). Evaluation of Interleukin-2 to detect active and latent tuberculosis among household contacts of pulmonary tuberculosis cases. Arch. Pediatr. Infect. Dis..

[139] Tan Y., Tan Y., Li J., Hu P., Guan P., Kuang H., Liang Q., Yu Y., Chen Z., Wang Q. (2021). Combined IFN-γ and IL-2 release assay for detect active pulmonary tuberculosis: A prospective multicentre diagnostic study in China. J. Transl. Med..

[140] Zhou Y., Zhang F., Shi H., Wu P. (2022). Host biomarkers other than interferon gamma in QFT-TB supernatants for identifying active tuberculosis. Tuberculosis.

[141] Santin M., Morandeira-Rego F., Alcaide F., Rabunal R., Anibarro L., Aguero-Balbin R., Casas-Garcia X., Perez-Escolano E., Navarro M.D., Sanchez F. (2016). Detection of interleukin-2 is not useful for distinguishing between latent and active tuberculosis in clinical practice: A prospective cohort study. Clin. Microbiol. Infect..

[142] Biselli R., Mariotti S., Sargentini V., Sauzullo I., Lastilla M., Mengoni F., Vanini V., Girardi E., Goletti D., D’ Amelio R. (2010). Detection of interleukin-2 in addition to interferon-γ discriminates active tuberculosis patients, latently infected individuals, and controls. Clin. Microbiol. Infect..

[143] Sun Q., Wei W., Sha W. (2016). Potential Role for Mycobacterium tuberculosis specific IL-2 and IFN-γ responses in discriminating between latent infection and active disease after long-term stimulation. PLoS ONE.

[144] Sargentini V., Mariotti S., Carrara S., Gagliardi M.C., Teloni R., Goletti D., Nisini R. (2009). Cytometric detection of antigen-specific IFN-γ/IL-2 secreting cells in the diagnosis of tuberculosis. BMC Infect. Dis..

[145] Gourgouillon N., de Lauzanne A., Cottart C.-H., Curis E., Debord C., Guérin-El Khourouj V., Pédron B., Faye A., Sterkers G. (2012). TNF-α/IL-2 ratio discriminates latent from active tuberculosis in immunocompetent children: A pilot study. Pediatr. Res..

[146] Wei Z., Li Y., Wei C., Li Y., Xu H., Wu Y., Jia Y., Guo R., Jia J., Qi X. (2020). The meta-analysis for ideal cytokines to distinguish the latent and active TB infection. BMC Pulm. Med..

[147] Qiu X., Wang H., Tang Y., Su X., Ge L., Qu Y., Mu D. (2020). Is interleukin-2 an optimal marker for diagnosing tuberculosis infection? A systematic review and meta-analysis. Ann. Med..

[148] Liu M., Guo S., Hibbert J.M., Jain V., Singh N., Wilson N.O., Stiles J.K. (2011). CXCL10/IP-10 in infectious diseases pathogenesis and potential therapeutic implications. Cytokine Growth Factor. Rev..

[149] Whittaker E., Gordon A., Kampmann B. (2008). Is IP-10 a better biomarker for active and latent tuberculosis in children than IFNγ?. PLoS ONE.

[150] Hong J.Y., Jung G.S., Kim H., Kim Y.M., Lee H.J., Cho S.-N., Kim S.K., Chang J., Kang Y.A. (2012). Efficacy of inducible protein 10 as a biomarker for the diagnosis of tuberculosis. Int. J. Infect. Dis..

[151] Petrone L., Vanini V., Chiacchio T., Petruccioli E., Cuzzi G., Schininà V., Palmieri F., Ippolito G., Goletti D. (2018). Evaluation of IP-10 in Quantiferon-Plus as biomarker for the diagnosis of latent tuberculosis infection. Tuberculosis.

[152] Estévez O., Anibarro L., Garet E., Pallares Á., Pena A., Villaverde C., Del Campo V., González-Fernández Á. (2020). Identification of candidate host serum and saliva biomarkers for a better diagnosis of active and latent tuberculosis infection. PLoS ONE.

[153] Lighter J., Rigaud M., Huie M., Peng C.H., Pollack H. (2009). Chemokine IP-10: An adjunct marker for latent tuberculosis infection in children. Int. J. Tuberc. Lung Dis..

[154] Blauenfeldt T., Villar-Hernández R., García-García E., Latorre I., Holm Line L., Muriel-Moreno B., De Souza-Galvão Maria L., Millet Joan P., Sabriá F., Sánchez-Montalva A. (2020). Diagnostic accuracy of interferon gamma-induced protein 10 mRNA release assay for tuberculosis. J. Clin. Microbiol..

[155] Wergeland I., Assmus J., Dyrhol-Riise A.M. (2016). Cytokine patterns in tuberculosis infection; IL-1ra, IL-2 and IP-10 differentiate borderline QuantiFERON-TB samples from uninfected controls. PLoS ONE.

[156] Uzorka J.W., Bakker J.A., van Meijgaarden K.E., Leyten E.M.S., Delfos N.M., Hetem D.J., Kerremans J., Zwarts M., Cozijn S., Ottenhoff T.H.M. (2022). Biomarkers to identify Mycobacterium tuberculosis infection among borderline QuantiFERON results. Eur. Respir. J..

[157] Qiu X., Tang Y., Zou R., Zeng Y., Yue Y., Li W., Qu Y., Mu D. (2019). Diagnostic accuracy of interferon-gamma-induced protein 10 for differentiating active tuberculosis from latent tuberculosis: A meta-analysis. Sci. Rep..

[158] Qiu X., Xiong T., Su X., Qu Y., Ge L., Yue Y., Zeng Y., Li W., Hu P., Mu D. (2019). Accumulate evidence for IP-10 in diagnosing pulmonary tuberculosis. BMC Infect. Dis..

[159] Qiu X., Tang Y., Yue Y., Zeng Y., Li W., Qu Y., Mu D. (2019). Accuracy of interferon-γ-induced protein 10 for diagnosing latent tuberculosis infection: A systematic review and meta-analysis. Clin. Microbiol. Infect..

[160] Zimmer A.J., Lainati F., Aguilera Vasquez N., Chedid C., McGrath S., Benedetti A., MacLean E., Ruhwald M., Denkinger C.M., Kohli M. (2022). Biomarkers that correlate with active pulmonary tuberculosis treatment response: A systematic review and meta-analysis. J. Clin. Microbiol..

[161] Parameswaran N., Patial S. (2010). Tumor necrosis factor-α signaling in macrophages. Crit. Rev. Eukaryot Gene Expr..

[162] Wang F., Hou H., Xu L., Jane M., Peng J., Lu Y., Zhu Y., Sun Z. (2013). Mycobacterium tuberculosis-specific TNF-α is a potential biomarker for the rapid diagnosis of active tuberculosis disease in Chinese population. PLoS ONE.

[163] Harari A., Rozot V., Bellutti Enders F., Perreau M., Stalder J.M., Nicod L.P., Cavassini M., Calandra T., Blanchet C.L., Jaton K. (2011). Dominant TNF-α+ Mycobacterium tuberculosis-specific CD4+ T cell responses discriminate between latent infection and active disease. Nat. Med..

[164] Zhang L., Wan S., Zhou Z., Zhang Y., Liu X. (2021). Utility of interferon gamma/tumor necrosis factor alpha FluoroSpot assay in differentiation between active tuberculosis and latent tuberculosis infection: A pilot study. BMC Infect. Dis..

[165] Kim J.Y., Kang Y.A., Park J.H., Cha H.H., Jeon N.Y., Lee S.W., Lee S.O., Choi S.H., Kim Y.S., Woo J.H. (2020). An IFN-γ and TNF-α dual release fluorospot assay for diagnosing active tuberculosis. Clin. Microbiol. Infect..

[166] Kim S., Lee H., Kim H., Kim Y., Cho J.-E., Jin H., Kim D.Y., Ha S.-J., Kang Y.A., Cho S.-N. (2015). Diagnostic performance of a cytokine and IFN-γ induced chemokine mRNA assay after Mycobacterium tuberculosis specific antigen stimulation in whole blood from infected individuals. J. Mol. Diagn..

[167] Prabhavathi M., Pathakumari B., Raja A. (2015). IFN-γ/TNF-α ratio in response to immuno proteomically identified human T-cell antigens of Mycobacterium tuberculosis—The most suitable surrogate biomarker for latent TB infection. J. Infect..

[168] Couper K.N., Blount D.G., Riley E.M. (2008). IL-10: The master regulator of immunity to infection. J. Immunol..

[169] Bapat P.R., Husain A.A., Daginawala H.F., Agrawal N.P., Panchbhai M.S., Satav A.R., Taori G.M., Kashyap R.S. (2015). The assessment of cytokines in Quantiferon supernatants for the diagnosis of latent TB infection in a tribal population of Melghat, India. J. Infect. Public Health.

[170] Lohela M., Bry M., Tammela T., Alitalo K. (2009). VEGFs and receptors involved in angiogenesis versus lymphangiogenesis. Curr. Opin. Cell Biol..

[171] Delemarre E.M., van Hoorn L., Bossink A.W.J., Drylewicz J., Joosten S.A., Ottenhoff T.H.M., Akkerman O.W., Goletti D., Petruccioli E., Navarra A. (2021). Serum biomarker profile including CCL1, CXCL10, VEGF, and adenosine deaminase activity distinguishes active from remotely acquired latent tuberculosis. Front. Immunol..

[172] Kumar N.P., Banurekha V.V., Nair D., Babu S. (2016). Circulating angiogenic factors as biomarkers of disease severity and bacterial burden in pulmonary tuberculosis. PLoS ONE.

[173] Rensburg I.C.v., Loxton A.G. (2015). Transcriptomics: The key to biomarker discovery during tuberculosis?. Biomark. Med..

[174] Petrilli J.D., Araújo L.E., da Silva L.S., Laus A.C., Müller I., Reis R.M., Netto E.M., Riley L.W., Arruda S., Queiroz A. (2020). Whole blood mRNA expression-based targets to discriminate active tuberculosis from latent infection and other pulmonary diseases. Sci. Rep..

[175] de Araujo L.S., Vaas L.A.I., Ribeiro-Alves M., Geffers R., Mello F.C.Q., de Almeida A.S., Moreira A.d.S.R., Kritski A.L., Lapa E Silva J.R., Moraes M.O. (2016). Transcriptomic biomarkers for tuberculosis: Evaluation of DOCK9. EPHA4, and NPC2 mRNA expression in peripheral blood. Front. Microbiol..

[176] Hashimoto S., Zhao H., Hayakawa M., Nakajima K., Taguchi Y.h., Murakami Y. (2020). Developing a diagnostic method for latent tuberculosis infection using circulating miRNA. Transl. Med. Commun..

[177] Wang C., Yang S., Sun G., Tang X., Lu S., Neyrolles O., Gao Q. (2011). Comparative miRNA expression profiles in individuals with latent and active tuberculosis. PLoS ONE.

[178] Wu L.S.-H., Lee S.-W., Huang K.-Y., Lee T.-Y., Hsu P.W.-C., Weng J.T.-Y. (2014). Systematic expression profiling analysis identifies specific microRNA-gene interactions that may differentiate between active and latent tuberculosis infection. BioMed Res. Int..

[179] Hamada Y., Penn-Nicholson A., Krishnan S., Cirillo D.M., Matteelli A., Wyss R., Denkinger C.M., Rangaka M.X., Ruhwald M., Schumacher S.G. (2022). Are mRNA based transcriptomic signatures ready for diagnosing tuberculosis in the clinic?—A review of evidence and the technological landscape. EBioMedicine.

[180] Araujo L.S.d., Ribeiro-Alves M., Leal-Calvo T., Leung J., Durán V., Samir M., Talbot S., Tallam A., Mello F.C.d.Q., Geffers R. (2019). Reprogramming of small noncoding RNA populations in peripheral blood reveals host biomarkers for latent and active Mycobacterium tuberculosis infection. mBio.

[181] Zak D.E., Penn-Nicholson A., Scriba T.J., Thompson E., Suliman S., Amon L.M., Mahomed H., Erasmus M., Whatney W., Hussey G.D. (2016). A blood RNA signature for tuberculosis disease risk: A prospective cohort study. Lancet.

[182] Darboe F., Mbandi S.K., Thompson E.G., Fisher M., Rodo M., van Rooyen M., Filander E., Bilek N., Mabwe S., Hatherill M. (2018). Diagnostic performance of an optimized transcriptomic signature of risk of tuberculosis in cryopreserved peripheral blood mononuclear cells. Tuberculosis.

[183] Scriba T.J., Fiore-Gartland A., Penn-Nicholson A., Mulenga H., Kimbung Mbandi S., Borate B., Mendelsohn S.C., Hadley K., Hikuam C., Kaskar M. (2021). Biomarker-guided tuberculosis preventive therapy (CORTIS): A randomised controlled trial. Lancet Infect. Dis..

[184] Mulenga H., Fiore-Gartland A., Mendelsohn S.C., Penn-Nicholson A., Mbandi S.K., Nemes E., Borate B., Musvosvi M., Tameris M., Walzl G. (2022). Evaluation of a transcriptomic signature of tuberculosis risk in combination with an interferon gamma release assay: A diagnostic test accuracy study. eClinicalMedicine.

[185] Estévez O., Anibarro L., Garet E., Pallares Á., Barcia L., Calviño L., Maueia C., Mussá T., Fdez-Riverola F., Glez-Peña D. (2020). An RNA-seq based machine learning approach identifies latent tuberculosis patients with an active tuberculosis profile. Front. Immunol..

[186] Phetsouphanh C., Zaunders J.J., Kelleher A.D. (2015). Detecting antigen-specific T cell responses: From bulk populations to single cells. Int. J. Mol. Sci..

[187] Pollock K.M., Whitworth H.S., Montamat-Sicotte D.J., Grass L., Cooke G.S., Kapembwa M.S., Kon O.M., Sampson R.D., Taylor G.P., Lalvani A. (2013). T-cell immunophenotyping distinguishes active from latent tuberculosis. J. Infect. Dis..

[188] Silveira-Mattos P.S., Barreto-Duarte B., Vasconcelos B., Fukutani K.F., Vinhaes C.L., Oliveira-De-Souza D., Ibegbu C.C., Figueiredo M.C., Sterling T.R., Rengarajan J. (2020). Differential expression of activation markers by Mycobacterium tuberculosis-specific CD4+ T cell distinguishes extrapulmonary from pulmonary tuberculosis and latent infection. Clin. Infect. Dis..

[189] Mpande C.A.M., Rozot V., Mosito B., Musvosvi M., Dintwe O.B., Bilek N., Hatherill M., Scriba T.J., Nemes E. (2021). Immune profiling of Mycobacterium tuberculosis-specific T cells in recent and remote infection. EBioMedicine.

[190] Ubolyam S., Iampornsin T., Sophonphan J., Avihingsanon A., Suwanpimolkul G., Kawkitinarong K., Manosuthi W., Gatechompol S., Ananworanich J., Ruxrungtham K. (2021). Performance of a simple flow cytometric assay in diagnosing active tuberculosis. Tuberculosis.

[191] Escalante P., Peikert T., Van Keulen V.P., Erskine C.L., Bornhorst C.L., Andrist B.R., McCoy K., Pease L.R., Abraham R.S., Knutson K.L. (2015). Combinatorial immunoprofiling in latent tuberculosis infection. toward better risk stratification. Am. J. Respir. Crit. Care Med..

[192] Estevez O., Anibarro L., Garet E., Martínez A., Pena A., Barcia L., Peleteiro M., Fernández Á. (2020). Multi-parameter flow cytometry immunophenotyping distinguishes different stages of tuberculosis infection. J. Infect..

[193] Duncan C., Jamieson F., Mehaffy C. (2015). Preliminary evaluation of exome sequencing to identify genetic markers of susceptibility to tuberculosis disease. BMC Res. Notes.

[194] Wu L., Hu Y., Li D., Jiang W., Xu B. (2015). Screening toll-like receptor markers to predict latent tuberculosis infection and subsequent tuberculosis disease in a Chinese population. BMC Med. Genet..

[195] Wu S., Liu X., Wang Y., Zhang M., Wang M., He J.-Q. (2019). Genetic polymorphisms of IFNG and IFNGR1 with latent tuberculosis infection. Dis. Markers.

[196] Hu Y., Wu L., Li D., Zhao Q., Jiang W., Xu B. (2015). Association between cytokine gene polymorphisms and tuberculosis in a Chinese population in Shanghai: A case–control study. BMC Immunol..

[197] Chang S.-Y., Chen M.-L., Lee M.-R., Liang Y.-C., Lu T.-P., Wang J.-Y., Yan B.-S. (2018). SP110 polymorphisms are genetic markers for vulnerability to latent and active tuberculosis infection in Taiwan. Dis. Markers.

[198] Cazarez-Navarro G., Palomares-Marín J., Rodríguez-Preciado S.Y., Pereira-Suárez A.L., Martínez-López E., Bacilio-Medrano E.A., Huerta-OlveraIván S., Hernández-Cañaveral I. (2021). Association of TAP1 1177A>G and 2090A>G gene polymorphisms with latent tuberculosis infections in sheltered populations, in the metropolitan area of Guadalajara, Mexico: A pilot study. Rev. Inst. Med. Trop São Paulo.

[199] Cubillos-Angulo J.M., Arriaga M.B., Melo M.G.M., Silva E.C., Alvarado-Arnez L.E., de Almeida A.S., Moraes M.O., Moreira A.S.R., Lapa e Silva J.R., Fukutani K.F. (2020). Polymorphisms in interferon pathway genes and risk of Mycobacterium tuberculosis infection in contacts of tuberculosis cases in Brazil. Int. J. Infect. Dis..

[200] Zhang S., Li G., Bi J., Guo Q., Fu X., Wang W., Liu S., Xiao G., Ou M., Zhang J. (2022). Association between functional nucleotide polymorphisms up-regulating transforming growth factor β1 expression and increased tuberculosis susceptibility. J. Infect. Dis..

[201] Teklu T., Wondale B., Taye B., Hailemariam M., Bekele S., Tamirat M., Zewude A., Mohamed T., Medhin G., Legesse M. (2020). Differences in plasma proteomes for active tuberculosis, latent tuberculosis and non-tuberculosis mycobacterial lung disease patients with and without ESAT-6/CFP10 stimulation. Proteome Sci..

[202] Liu L., Deng J., Yang Q., Wei C., Liu B., Zhang H., Xin H., Pan S., Liu Z., Wang D. (2021). Urinary proteomic analysis to identify a potential protein biomarker panel for the diagnosis of tuberculosis. IUBMB Life.

[203] Deng J., Liu L., Yang Q., Wei C., Zhang H., Xin H., Pan S., Liu Z., Wang D., Liu B. (2021). Urinary metabolomic analysis to identify potential markers for the diagnosis of tuberculosis and latent tuberculosis. Arch. Biochem. Biophys..

[204] Mateos J., Estévez O., González-Fernández Á., Anibarro L., Pallarés Á., Reljic R., Gallardo J.M., Medina I., Carrera M. (2019). High-resolution quantitative proteomics applied to the study of the specific protein signature in the sputum and saliva of active tuberculosis patients and their infected and uninfected contacts. J. Proteomics.

[205] Mateos J., Estévez O., González-Fernández Á., Anibarro L., Pallarés Á., Reljic R., Mussá T., Gomes-Maueia C., Nguilichane A., Gallardo J.M. (2020). Serum proteomics of active tuberculosis patients and contacts reveals unique processes activated during Mycobacterium tuberculosis infection. Sci. Rep..

[206] Serra-Vidal M.M., Latorre I., Franken K.L.C.M., Díaz J., de Souza-Galvão M.L., Casas I., Maldonado J., Milà C., Solsona J., Jimenez-Fuentes M.Á. (2014). Immunogenicity of 60 novel latency-related antigens of Mycobacterium tuberculosis. Front. Microbiol..

[207] Leyten E.M.S., Lin M.Y., Franken K.L.M.C., Friggen A.H., Prins C., van Meijgaarden K.E., Voskuil M.I., Weldingh K., Andersen P., Schoolnik G.K. (2006). Human T-cell responses to 25 novel antigens encoded by genes of the dormancy regulon of Mycobacterium tuberculosis. Microbes Infect..

[208] Zhang L., Ma H., Wan S., Zhang Y., Gao M., Liu X. (2022). Mycobacterium tuberculosis latency-associated antigen Rv1733c SLP improves the accuracy of differential diagnosis of active tuberculosis and latent tuberculosis infection. Chin. Med. J..

[209] Arroyo L., Marín D., Franken K.L.M.C., Ottenhoff T.H.M., Barrera L.F. (2018). Potential of DosR and Rpf antigens from Mycobacterium tuberculosis to discriminate between latent and active tuberculosis in a tuberculosis endemic population of Medellin Colombia. BMC Infect. Dis..

[210] Goletti D., Butera O., Vanini V., Lauria F.N., Lange C., Franken K.L.M.C., Angeletti C., Ottenhoff T.H.M., Girardi E. (2010). Response to Rv2628 latency antigen associates with cured tuberculosis and remote infection. Eur. Respir. J..

[211] Adankwah E., Nausch N., Minadzi D., Abass M.K., Franken K.L.M.C., Ottenhoff T.H.M., Mayatepek E., Phillips R.O., Jacobsen M. (2021). Interleukin-6 and Mycobacterium tuberculosis dormancy antigens improve diagnosis of tuberculosis. J. Infect..

[212] Peña D., Rovetta A.I., Hernández Del Pino R.E., Amiano N.O., Pasquinelli V., Pellegrini J.M., Tateosian N.L., Rolandelli A., Gutierrez M., Musella R.M. (2015). A Mycobacterium tuberculosis dormancy antigen differentiates latently infected Bacillus Calmette–Guérin-vaccinated individuals. EBioMedicine.

[213] Amiano N.O., Morelli M.P., Pellegrini J.M., Tateosian N.L., Rolandelli A., Seery V., Castello F.A., Gallego C., Armitano R., Stupka J. (2020). IFN-γ and IgG responses to Mycobacterium tuberculosis latency antigen Rv2626c differentiate remote from recent tuberculosis infection. Sci. Rep..

[214] Jee B., Singh Y., Yadav R., Lang F. (2018). Small heat shock protein16.3 of Mycobacterium tuberculosis: After two decades of functional characterization. Cell Physiol. Biochem..

[215] Belay M., Legesse M., Mihret A., Bekele Y., Ottenhoff T.H.M., Franken K.L.M.C., Bjune G., Abebe F. (2015). Pro- and anti-inflammatory cytokines against Rv2031 are elevated during latent tuberculosis: A study in cohorts of tuberculosis patients, household contacts and community controls in an endemic setting. PLoS ONE.

[216] Demissie A., Leyten E.M.S., Abebe M., Wassie L., Aseffa A., Abate G., Fletcher H., Owiafe P., Hill P.C., Brookes R. (2006). Recognition of stage-specific mycobacterial antigens differentiates between acute and latent infections with Mycobacterium tuberculosis. Clin. Vaccine Immunol..

[217] Doddam S.N., Peddireddy V., Ahmed N. (2017). Mycobacterium tuberculosis DosR regulon gene Rv2004c encodes a novel antigen with pro-inflammatory functions and potential diagnostic application for detection of latent tuberculosis. Front. Immunol..

[218] Huang W., Qi Y., Ren C., Wen H., Franken K.L.M.C., Ottenhoff T.H.M., Shen J. (2013). Interferon-γ responses to Mycobacterium tuberculosis Rpf proteins in contact investigation. Tuberculosis.

[219] Wang S., Wu J., Chen J., Gao Y., Zhang S., Zhou Z., Huang H., Shao L., Jin J., Zhang Y. (2018). Evaluation of Mycobacterium tuberculosis-specific antibody responses for the discrimination of active and latent tuberculosis infection. Int. J. Infect. Dis..

[220] WHO (2011). WHO Warns against the Use of Inaccurate Blood Tests for Active Tuberculosis.

[221] Li Z., Hu J., Liu P., Cui D., Di H., Wu S. (2021). Microarray-based selection of a serum biomarker panel that can discriminate between latent and active pulmonary TB. Sci. Rep..

[222] Kumar S.K., Arya S., Aggarwal A., Kapoor P., Nath A., Misra R., Sinha S. (2020). Immune responses to Mycobacterium tuberculosis membrane-associated antigens including alpha crystallin can potentially discriminate between latent infection and active tuberculosis disease. PLoS ONE.

[223] Castro-Garza J., García-Jacobo P., Rivera-Morales L.G., Quinn F.D., Barber J., Karls R., Haas D., Helms S., Gupta T., Blumberg H. (2017). Detection of anti-HspX antibodies and HspX protein in patient sera for the identification of recent latent infection by Mycobacterium tuberculosis. PLoS ONE.

[224] Rajpal S.K., Snehal S.W., Miling S.P., Hemant J.P., Girdhar M.T., Hatim F.D. (2011). Mycobacterium tuberculosis heat shock protein 16 as a potential marker for latent TB: A preliminary findings. J. Clin. Cell Immunol..

[225] Coppola M., Arroyo L., van Meijgaarden K.E., Franken K.L.M.C., Geluk A., Barrera L.F., Ottenhoff T.H.M. (2017). Differences in IgG responses against infection phase related Mycobacterium tuberculosis (Mtb) specific antigens in individuals exposed or not to Mtb correlate with control of TB infection and progression. Tuberculosis.

[226] Kasempimolporn S., Thaveekarn W., Promrungreang K., Khow O., Boonchang S., Sitprija V. (2017). Improved serodiagnostic sensitivity of strip test for latent tuberculosis. J. Clin. Diagn. Res..

[227] Zhang C., Song X., Zhao Y., Zhang H., Zhao S., Mao F., Bai B., Wu S., Shi C. (2015). Mycobacterium tuberculosis secreted proteins as potential biomarkers for the diagnosis of active tuberculosis and latent tuberculosis infection. J. Clin. Lab. Anal..

[228] Lee J.Y., Kim B.-J., Koo H.-K., Kim J., Kim J.-M., Kook Y.-H., Kim B.-J. (2020). Diagnostic potential of IgG and IgA responses to Mycobacterium tuberculosis antigens for discrimination among active tuberculosis, latent tuberculosis infection, and non-infected individuals. Microorganisms.

[229] Maekura R., Kitada S., Osada-Oka M., Tateishi Y., Ozeki Y., Fujicawa T., Miki M., Jyunnko O., Mori M., Matsumoto S. (2019). Serum antibody profiles in individuals with latent Mycobacterium tuberculosis infection. Microbiol. Immunol..

[230] Pan S.-W., Su W.-J., Chan Y.-J., Chuang F.-Y., Feng J.-Y., Chen Y.-M. (2021). Mycobacterium tuberculosis–derived circulating cell-free DNA in patients with pulmonary tuberculosis and persons with latent tuberculosis infection. PLoS ONE.

[231] Bajgai P., Sharma K., Bansal R., Gupta N., Sharma A., Gupta A. (2016). Detection of Mycobacterium tuberculosis genome in subretinal fluid of patients with latent tuberculosis infection. Ocul. Immunol. Inflamm..

[232] Das B., Kashino S.S., Pulu I., Kalita D., Swami V., Yeger H., Felsher D.W., Campos-Neto A. (2013). CD271(+) bone marrow mesenchymal stem cells may provide a niche for dormant Mycobacterium tuberculosis. Sci. Transl. Med..

[233] Belay M., Tulu B., Younis S., Jolliffe D.A., Tayachew D., Manwandu H., Abozen T., Tirfie E.A., Tegegn M., Zewude A. (2021). Detection of <em>Mycobacterium tuberculosis</em> complex DNA in CD34-positive peripheral blood mononuclear cells of asymptomatic tuberculosis contacts: An observational study. Lancet Microbe.

[234] Young B.L., Mlamla Z., Gqamana P.P., Smit S., Roberts T., Peter J., Theron G., Govender U., Dheda K., Blackburn J. (2014). The identification of tuberculosis biomarkers in human urine samples. Eur. Respir. J..

[235] Phan L.M.T., Kim E.B., Cheon S.A., Shim T.S., Kim H.-J., Park T.J. (2020). Reliable naked-eye detection of Mycobacterium tuberculosis antigen 85B using gold and copper nanoshell-enhanced immunoblotting techniques. Sens. Actuators B Chem..

[236] Napolitano D.R., Pollock N., Kashino S.S., Rodrigues V., Campos-Neto A. (2008). Identification of Mycobacterium tuberculosis ornithine carboamyltransferase in urine as a possible molecular marker of active pulmonary tuberculosis. Clin. Vaccine Immunol..

[237] Mehaffy C., Dobos K.M., Nahid P., Kruh-Garcia N.A. (2017). Second generation multiple reaction monitoring assays for enhanced detection of ultra-low abundance Mycobacterium tuberculosis peptides in human serum. Clin. Proteom..

[238] Mehaffy C., Kruh-Garcia Nicole A., Graham B., Jarlsberg Leah G., Willyerd Charis E., Borisov A., Sterling Timothy R., Nahid P., Dobos Karen M., Land Geoffrey A. (2020). Identification of Mycobacterium tuberculosis peptides in serum extracellular vesicles from persons with latent tuberculosis infection. J. Clin. Microbiol..

[239] WHO (2020). WHO Consolidated Guidelines on Tuberculosis: Tuberculosis Preventive Treatment: Module 1: Prevention.

[240] Scolarici M., Dekitani K., Chen L., Sokol-Anderson M., Hoft D.F., Chatterjee S. (2018). A scoring strategy for progression risk and rates of treatment completion in subjects with latent tuberculosis. PLoS ONE.

[241] Hesseling A.C., Mandalakas A.M., Kirchner H.L., Chegou N.N., Marais B.J., Stanley K., Zhu X., Black G., Beyers N., Walzl G. (2009). Highly discordant T cell responses in individuals with recent exposure to household tuberculosis. Thorax.

[242] Li R., Nordio F., Huang C.C., Contreras C., Calderon R., Yataco R., Galea J.T., Zhang Z., Becerra M.C., Lecca L. (2020). Two clinical prediction tools to improve tuberculosis contact investigation. Clin. Infect. Dis..

[243] Aksornchindarat W., Yodpinij N., Phetsuksiri B., Srisungngam S., Rudeeaneksin J., Bunchoo S., Klayut W., Sangkitporn S., Khawcharoenporn T. (2021). T-SPOT®.TB test and clinical risk scoring for diagnosis of latent tuberculosis infection among Thai healthcare workers. J. Microbiol. Immunol. Infect..

[244] WHO (2015). Guidelines on the Management of Latent Tuberculosis Infection.

[245] Dobler C.C., Martin A., Marks G.B. (2015). Benefit of treatment of latent tuberculosis infection in individual patients. Eur. Respir. J..

[246] CDC (2020). Treatment Regimens for Latent TB Infection (LTBI).

[247] Swindells S., Ramchandani R., Gupta A., Benson C.A., Leon-Cruz J., Mwelase N., Jean Juste M.A., Lama J.R., Valencia J., Omoz-Oarhe A. (2019). One month of rifapentine plus isoniazid to prevent HIV-related tuberculosis. N. Engl. J. Med..

[248] CDC (2022). Guidelines for the Prevention and Treatment of Opportunistic Infections in Adults and Adolescents with HIV.

[249] Pradipta I.S., Houtsma D., van Boven J.F.M., Alffenaar J.-W.C., Hak E. (2020). Interventions to improve medication adherence in tuberculosis patients: A systematic review of randomized controlled studies. NPJ Prim. Care Respir. Med..

[250] Anibarro L., Casas S., Paz-Esquete J., Gonzalez L., Pena A., Guerra M.R., Sande D., Calviño L., Santin M., SEIMC (2010). Treatment completion in latent tuberculosis infection at specialist tuberculosis units in Spain. Int. J. Tuberc. Lung Dis..

[251] Oren E., Bell M.L., Garcia F., Perez-Velez C., Gerald L.B. (2017). Promoting adherence to treatment for latent TB infection through mobile phone text messaging: Study protocol for a pilot randomized controlled trial. Pilot Feasibility Stud..

[252] Sterling T.R., Villarino M.E., Borisov A.S., Shang N., Gordin F., Bliven-Sizemore E., Hackman J., Hamilton C.D., Menzies D., Kerrigan A. (2011). Three months of rifapentine and isoniazid for latent tuberculosis infection. N. Engl. J. Med..

[253] Chung S.J., Lee H., Koo G.W., Min J.-H., Yeo Y., Park D.W., Park T.S., Moon J.-Y., Kim S.-H., Kim T.H. (2020). Adherence to nine-month isoniazid for latent tuberculosis infection in healthcare workers: A prospective study in a tertiary hospital. Sci. Rep..

[254] Lin S.-Y., Chiu Y.-W., Lu P.-L., Hwang S.-J., Chen T.-C., Hsieh M.-H., Chen Y.-H. (2019). Three months of rifapentine and isoniazid for latent tuberculosis infection in hemodialysis patients: High rates of adverse events. J. Microbiol. Immunol. Infect..

[255] Ilaiwy G., Dowdy D.W. (2021). Cost effectiveness of three months of rifapentine and isoniazid for latent tuberculosis in Syrian refugees. J. Clin. Tuberc. Other Mycobact Dis..

[256] Borisov A.S., Bamrah Morris S., Njie G.J., Winston C.A., Burton D., Goldberg S., Yelk Woodruff R., Allen L., LoBue P., Vernon A. (2018). Update of recommendations for use of once-weekly isoniazid-rifapentine regimen to treat latent mycobacterium tuberculosis infection. MMWR Morb. Mortal. Wkly. Rep..

[257] Malik A.A., Gandhi N.R., Lash T.L., Cranmer L.M., Omer S.B., Ahmed J.F., Siddiqui S., Amanullah F., Khan A.J., Keshavjee S. (2021). Effectiveness of preventive therapy for persons exposed at home to drug-resistant tuberculosis, Karachi, Pakistan. Emerg. Infect. Dis..

[258] Nahid P., Mase S.R., Migliori G.B., Sotgiu G., Bothamley G.H., Brozek J.L., Cattamanchi A., Cegielski J.P., Chen L., Daley C.L. (2019). Treatment of drug-resistant tuberculosis. An official ATS/CDC/ERS/IDSA clinical practice guideline. Am. J. Respir. Crit. Care Med..

[259] Ferguson O., Jo Y., Pennington J., Johnson K., Chaisson R.E., Churchyard G., Dowdy D. (2020). Cost-effectiveness of one month of daily isoniazid and rifapentine versus three months of weekly isoniazid and rifapentine for prevention of tuberculosis among people receiving antiretroviral therapy in Uganda. J. Int. AIDS Soc..

[260] Dobler C.C., Batbayar O., Wright C.M. (2018). Practical challenges and solutions to TB control in a lower-middle-income country: Experiences from Mongolia. Breathe.

[261] Horn D.L., Hewlett D., Alfalla C., Peterson S., Opal S.M. (1994). Limited tolerance of ofloxacin and pyrazinamide prophylaxis against tuberculosis. N. Engl. J. Med..

[262] Younossian A.B., Rochat T., Ketterer J.-P., Wacker J., Janssens J.-P. (2005). High hepatotoxicity of pyrazinamide and ethambutol for treatment of latent tuberculosis. Eur. Respir. J..

[263] Bamrah S., Brostrom R., Dorina F., Setik L., Song R., Kawamura L.M., Heetderks A., Mase S. (2014). Treatment for LTBI in contacts of MDR-TB patients, Federated States of Micronesia, 2009–2012. Int. J. Tuberc. Lung Dis..

[264] Fox G.J., Nguyen C.B., Nguyen T.A., Tran P.T., Marais B.J., Graham S.M., Nguyen B.H., Velen K., Dowdy D.W., Mason P. (2020). Levofloxacin versus placebo for the treatment of latent tuberculosis among contacts of patients with multidrug-resistant tuberculosis (the VQUIN MDR trial): A protocol for a randomised controlled trial. BMJ Open.

[265] Seddon J.A., Garcia-Prats A.J., Purchase S.E., Osman M., Demers A.-M., Hoddinott G., Crook A.M., Owen-Powell E., Thomason M.J., Turkova A. (2018). Levofloxacin versus placebo for the prevention of tuberculosis disease in child contacts of multidrug-resistant tuberculosis: Study protocol for a phase III cluster randomised controlled trial (TB-CHAMP). Trials.

[266] NIH (2021). Protecting Households on Exposure to Newly Diagnosed Index Multidrug-Resistant Tuberculosis Patients (PHOENIx MDR-TB). https://clinicaltrials.gov/ct2/show/NCT03568383.

